# Chloride Salts and Light Modulate Germination and Early Seedling Performance in *Dittrichia viscosa*

**DOI:** 10.3390/plants15172607

**Published:** 2026-08-26

**Authors:** María del Pilar Cordovilla, Isidro Sánchez, Marlene Gladys Aguilar, Manuel Melendo

**Affiliations:** 1 Institute of Research on Olive Groves and Olive Oils, University of Jaén, Campus Las Lagunillas, 23071 Jaén, Spain; 2 Department of Animal Biology, Plant Biology and Ecology, Faculty of Experimental Sciences, University of Jaén, Campus Las Lagunillas, 23071 Jaén, Spain; isc00038@red.ujaen.es (I.S.); mmelendo@ujaen.es (M.M.); 3 Department of Horticulture, National Agrarian University of La Molina, Lima 15024, Peru; maguilarhe@lamolina.edu.pe

**Keywords:** *Dittrichia viscosa*, chloride salts, light regime, germination recovery, seedling vigour, salt tolerance

## Abstract

Soil salinity is one of the main environmental factors limiting plant germination and establishment, particularly in Mediterranean ecosystems. This study evaluated the germination, recovery, and early development of *Dittrichia viscosa* exposed to NaCl, KCl, CaCl_2_, MgCl_2_, and an equimolar mixture (0–360 mM) under a 12 h light–dark regime and continuous darkness. In the absence of salt, final germination was similar under both regimes, although it was faster in the dark. Increased salt concentration reduced and delayed germination, depending on the type of salt and the light regime used. KCl maintained the highest germination percentages under the light–dark regime at 90 and 180 mM, whilst CaCl_2_ and MgCl_2_ were the most inhibitory individual salts at 180 mM. The salt mixture was the most restrictive under both regimes. Following transfer to a salt-free medium, ungerminated seeds showed a high rate of recovery even after exposure to the highest concentrations of individual salts, indicating that the inhibition was largely reversible. The first two components of the principal component analysis (PCA) explained 71.89% of the variation, linking germination and its rate to seedling vigour. Overall, *D. viscosa* showed considerable germination tolerance to individual salts but high sensitivity to their combined action.

## 1. Introduction

Soil salinity is one of the main abiotic factors that limit seed germination and seedling establishment and influence the distribution of plant species, particularly in Mediterranean coastal ecosystems, salt marshes, and arid areas [1,2,3]. Its impact is particularly significant in the Mediterranean basin, where irregular and reduced rainfall, high evapotranspiration, seawater intrusion, rising sea levels, and certain anthropogenic processes can promote salt accumulation [1,4,5]. During germination, salt stress reduces external water potential, delays imbibition and hinders metabolic reactivation; at the same time, the influx and accumulation of ions can alter enzymatic activity, membrane stability, and the nutritional balance of embryonic tissues and newly emerged seedlings [1,6,7,8]. Furthermore, salinised soils contain complex ionic solutions and are rarely composed exclusively of NaCl, as they may accumulate carbonates, bicarbonates, chlorides, and sulphates associated with Na^+^, K^+^, Ca^2+^, and Mg^2+^ in varying proportions. This ionic diversity alters the physicochemical properties of the environment and may differentially affect germination and early growth [1,7]. Comparative studies using different chloride salts have also shown that the germination response and early growth may depend on the nature of the salt and on whether salts are applied individually or in combination [7].

Germination is one of the most critical and salt-stress-sensitive stages of the plant life cycle and largely determines the success of population establishment in saline environments [2,9]. In many halophytes and salt-tolerant species, exposure to high salt concentrations reduces or delays germination without necessarily compromising seed viability [10,11]. Seeds may remain ungerminated whilst stress persists and only resume germination when rainfall or soil leaching reduces salt concentrations [10,11,12,13]. Recovery assays, in which ungerminated seeds are transferred to a non-saline medium, allow the proportion of seeds capable of germinating following the alleviation of stress to be quantified and the degree of reversibility of salt inhibition to be assessed [11,12]. This ability may favour the persistence of populations in environments subject to seasonal fluctuations in salinity by delaying germination during unfavourable periods and concentrating it following rainfall when conditions are more suitable for seedling emergence and establishment [10,12,13]. Although in some halophytes germination may be more sensitive to salinity than early vegetative growth, salt stress can also severely reduce radicle and hypocotyl elongation, as well as seedling biomass and vigour. Consequently, the tolerance observed during germination does not, on its own, allow us to infer the success of subsequent establishment [2,14,15,16]. The final germination percentage is a basic indicator of stress response, but it does not, on its own, describe the dynamics of the process, as treatments with similar final values may differ in the onset, rate, synchronisation, or temporal distribution of germination. Consequently, the combined analyses of different temporal indices allows us to characterise complementary components of the germination response and identify those most sensitive to salinity [17,18].

The light regime, together with temperature, water availability, and salinity, helps to define the germination niche and may therefore influence the microhabitats and time periods in which the emergence and recruitment of new seedlings occur [3,19]. In saline environments, light conditions can alter the extent of germination inhibition. Khan and Ungar [20] observed in several halophytes that the inhibitory effect of salinity was more pronounced in the dark than in the light, whilst Mesléard et al. [21] showed that the light regime modulated germination responses to salinity and temperature in Juncus acutus and Juncus maritimus. Similarly, in *Cistus monspeliensis*, although germination was generally considered to be aphotoblastic, the effect of certain salinity treatments differed between light and continuous darkness [7]. The germination response to light regime may also exhibit intraspecific variation associated with seed provenance, as populations of the same species may differ in their germination parameters and optima under different combinations of light, temperature and salinity [9,22]. Taken together, this evidence suggests that the interaction between the light regime and salinity can alter the environmental windows favourable for germination and, consequently, recruitment success.

In this context, *Dittrichia viscosa* (L.) Greuter, traditionally cited as *Inula viscosa* (L.) Aiton, is a perennial species of the family Asteraceae native to the Mediterranean region and Western Asia, as well as widely distributed across Southern Europe, Turkey, the Middle East, and North Africa [23,24,25]. In the Mediterranean basin, it occurs mainly in habitats such as abandoned fields, roadside verges, rubbish dumps, and other ruderal or anthropogenically disturbed habitats [24,25,26]. The species exhibits high ecological plasticity and tolerates adverse environmental conditions, including drought, disturbed soils, and certain levels of salinity, which enhances its ability to colonise and its presence in degraded or marginal environments [24,25,27]. These characteristics have led to its consideration for the control of desertification, revegetation, and phytoremediation of degraded soils [24,25,27]. Given that the early stages of the life cycle determine the establishment of populations in saline environments, germination response, and early seedling development are essential aspects for defining the species’ ecological and applied potential [2,26].

The available information on the response of *D. viscosa* to salinity during the early stages of its life cycle is limited and fragmentary. Previous studies have focused mainly on treatments with NaCl and have not compared the species’ response to salts of different compositions [26,28,29]. Most laboratory studies of salinity during germination have assessed individual salts, particularly NaCl. However, saline soils commonly contain several ions simultaneously; therefore, comparing individual chloride salts with their equimolar mixture provides a more realistic representation of the ionic complexity of saline environments than the use of a single salt alone [7]. Therefore, the objectives of this study were: (i) to evaluate the effects of the salt type, salt concentration, and light regime on the germination and initial growth of seedlings; (ii) to determine the capacity for recovery following the removal of salt stress; and (iii) to quantify Cl^−^ tolerance by estimating Cl^−^_max_ and collectively analyse the relationships between the germination, recovery, and growth parameters. To this end, NaCl, KCl, CaCl_2_, MgCl_2_, and an equimolar mixture of these salts were compared under a light–dark regime and in continuous darkness. The hypothesis was that the early response of *D. viscosa* would depend not only on salt concentration but also on ionic composition and light regime, and that an integrated analysis of the parameters assessed would enable the detection of differences in tolerance not reflected solely by the final germination percentage. This approach will enable a comprehensive characterisation of the species’ early tolerance and will contribute to an understanding of its ability to establish itself in saline environments.

## 2. Results

The statistical results are summarised in Table 1 and Table 2. Table 1 reports the analysis of deviance for the binomial generalised linear models fitted to the germination percentage at 14, 21, and 30 days (FGP_14_, FGP_21_, FGP_30_), as well as the total germination percentage after 30 d of salt treatment and 15 d of recovery (TGP_45_). Saline treatment significantly affected all four germination percentages. Both the light regime and light regime × saline treatment interaction significantly affected FGP_14_, FGP_21_, and FGP_30_, indicating that the response during salt exposure depended on light conditions. In contrast, saline treatment remained significant for TGP_45_, whereas neither the light regime nor its interaction with saline treatment had a significant effect. Table 2 presents the results of the three-factor ANOVA on the effects of the light regime, salt type, salt concentration, and their interactions on the remaining germination and recovery parameters of *Dittrichia viscosa*. The salt type, salt concentration, and their interaction significantly affected all the parameters assessed, indicating that the effect of increasing concentration depended on the salt type. The light regime × salt type interaction was significant only for the mean germination time (MGT), time to 50% of final germination (T_50_), uncertainty (U), recovery percentage (RP), and mean germination time during recovery (MGT*_recovery_*). The two-factor ANOVAs carried out separately for each light regime showed that the salt type × salt concentration interaction was significant for all parameters, both under the light–dark regime and continuous darkness (Supplementary Table S1).

### 2.1. Effects of Salt Stress on Seed Germination and Recovery

**Figure 1:**
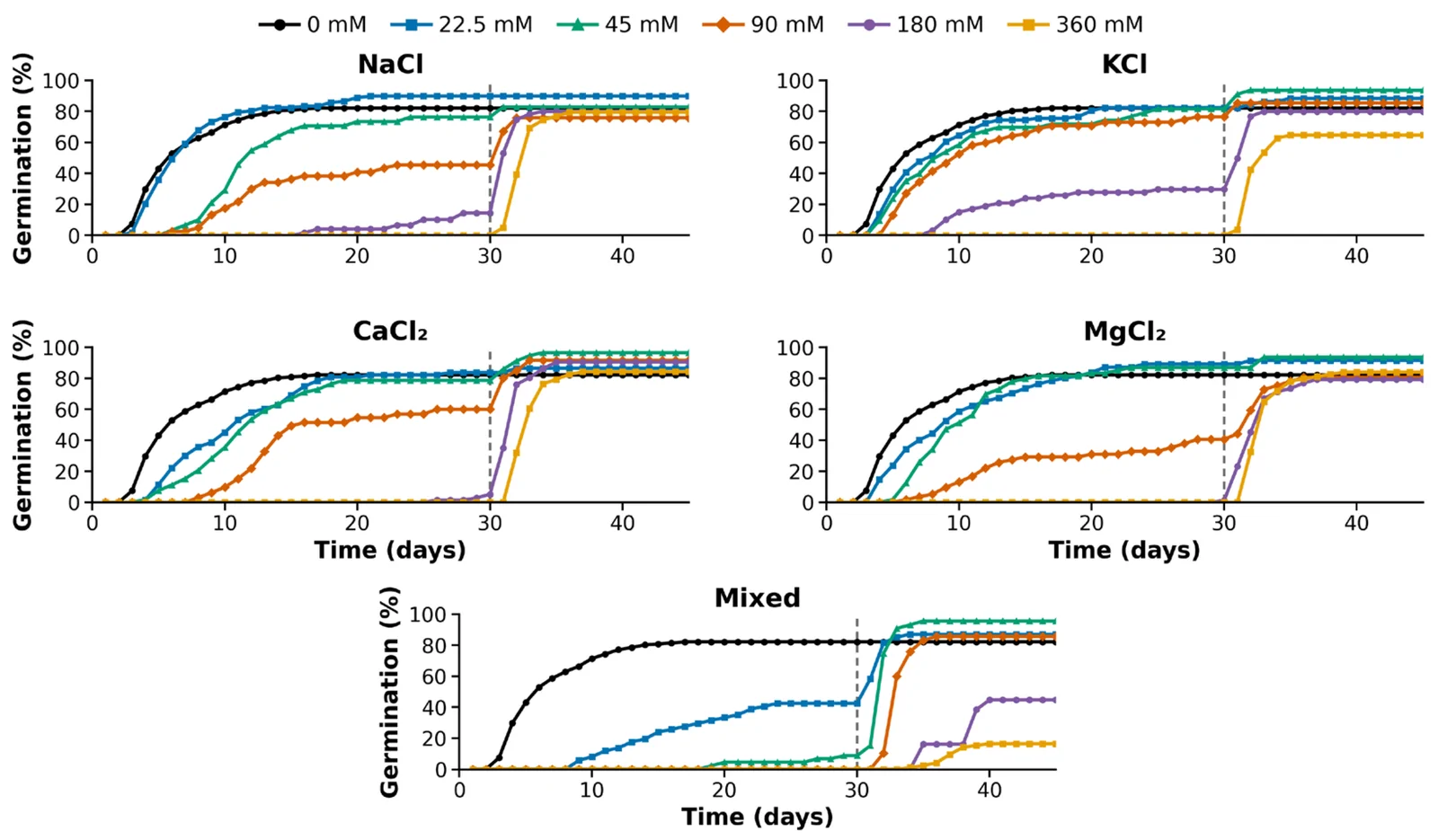
Cumulative germination of *D. viscosa* seeds under different salt types and concentrations in the light–dark regime. The vertical dashed line indicates transfer to distilled water after 30 days of saline treatment and the beginning of the recovery period.

**Figure 2:**
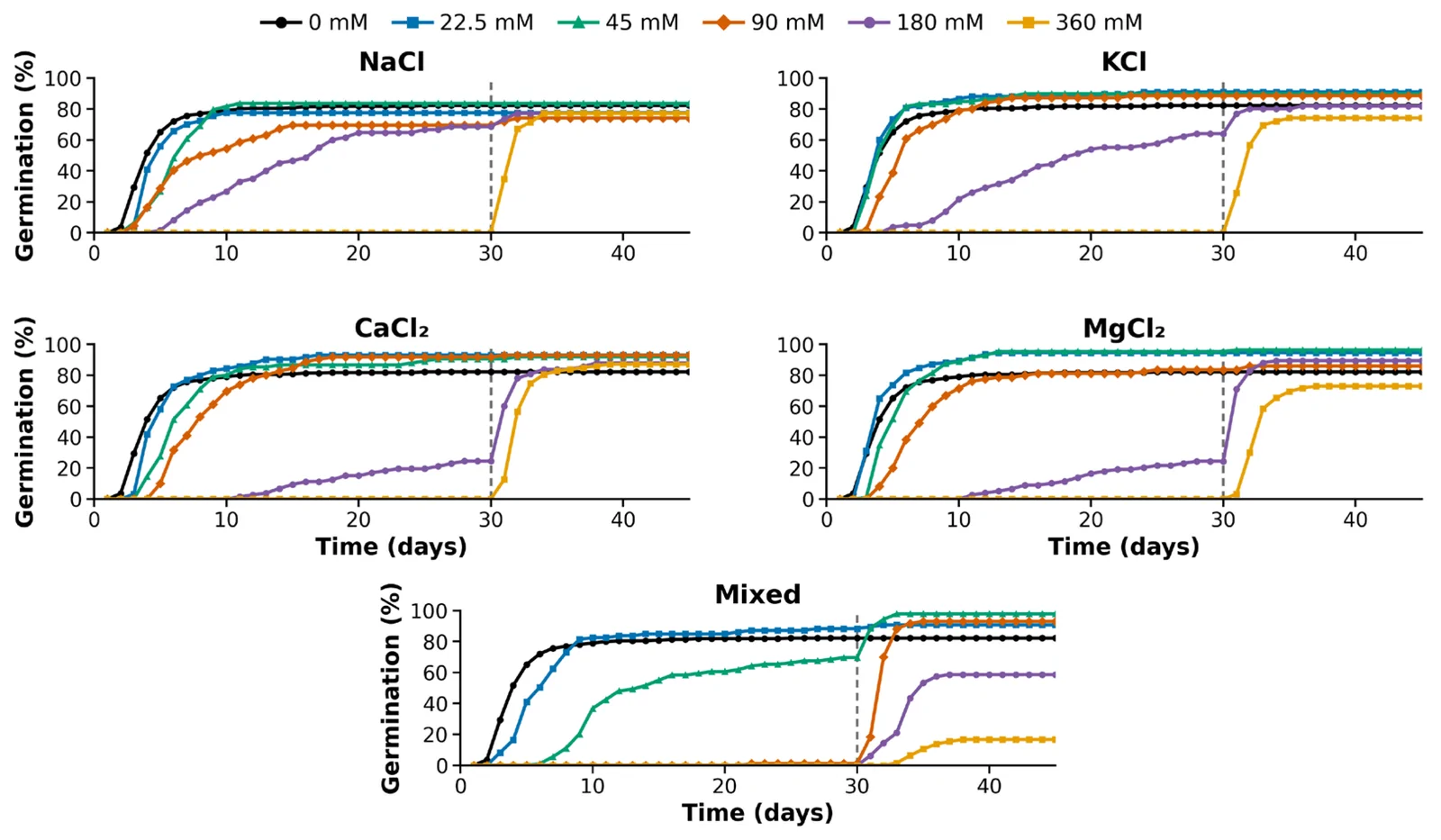
Cumulative germination of *D. viscosa* seeds under different salt types and concentrations in continuous darkness. The vertical dashed line indicates transfer to distilled water after 30 days of saline treatment and the beginning of the recovery period.

The cumulative germination curves showed differences between treatments over the 30-day period of salt exposure (Figure 1 and Figure 2). In general, higher concentrations delayed germination and reduced the cumulative percentage at the end of this phase, although the magnitude of these effects depended on the type of salt and light regime. The delay observed in the curves was reflected in the overall decrease in FGP_14_ and FGP_21_ (Supplementary Table S2). Inhibition was more pronounced under the light–dark regime and with the salt mixture.

**Figure 3:**
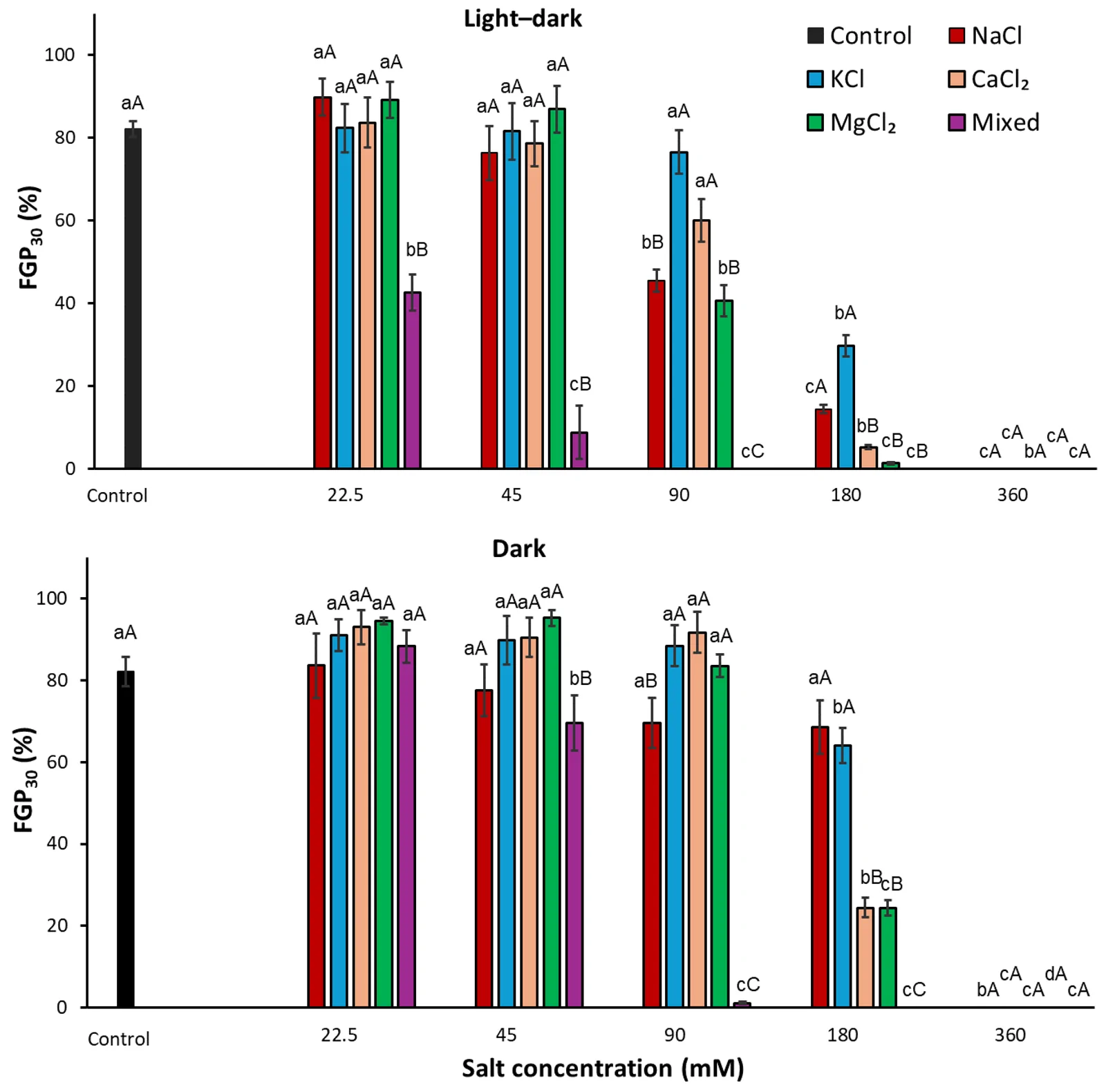
Final germination percentage after 30 days (FGP_30_) of *D. viscosa* seeds exposed to increasing concentrations of NaCl, KCl, CaCl_2_, MgCl_2_, and a salt mixture under the light–dark regime and continuous darkness. Bars represent means ± standard error (SE). Different lowercase letters indicate significant differences among concentrations within each salt, whilst different uppercase letters indicate significant differences among salts at the same concentration (Tukey-adjusted pairwise binomial comparisons, *p* < 0.05). The 0 mM treatment corresponds to the control.

For seeds not treated with salt, the final germination percentage was approximately 82% under both light regimes, with no significant differences between them (Figure 3). In the case of the individual salts, the differences between the light regimes became particularly evident at concentrations of 90 mM and above. Under the light–dark regime, KCl and CaCl_2_ maintained FGP_30_ values that did not differ significantly from the control, reaching 76.48% and 60.02%, respectively, whereas NaCl and MgCl_2_ reached 45.42% and 40.63%, respectively. Under continuous darkness, KCl, CaCl_2_, and MgCl_2_ did not differ significantly from the control at 90 mM, whereas NaCl showed a significantly lower FGP_30_ value (69.53%). At 180 mM, NaCl and KCl showed the highest values under both regimes. Their corresponding values were 14.40% and 29.71% under the light–dark regime, and 68.56% and 64.06% under continuous darkness. With the salt mixture, FGP_30_ decreased significantly from 22.5 mM under the light–dark regime, and germination was completely inhibited from 90 mM, whilst under continuous darkness, complete inhibition occurred from 180 mM. At 360 mM, no germination was recorded during the salt exposure phase in any treatment, regardless of the light regime (Figure 1, Figure 2 and Figure 3).

**Figure 4:**
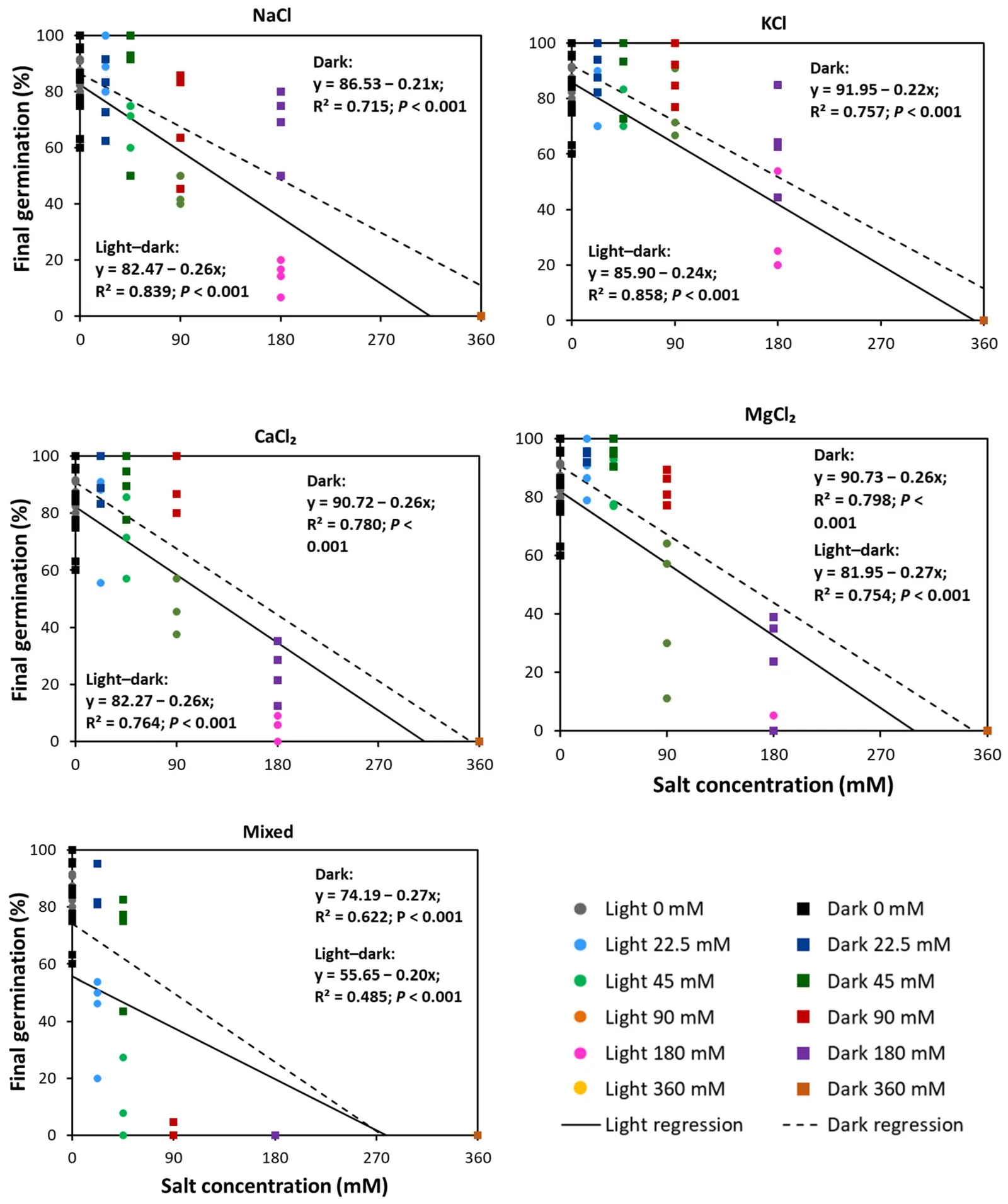
Linear regressions between salt concentration and final germination percentage at 30 days (FGP_30_) of *D. viscosa* seeds under the light–dark regime and continuous darkness.

Linear regression analysis confirmed the decrease in FGP_30_ with increasing salt concentration observed in the cumulative germination curves (*p* < 0.001; Figure 4). Under the light–dark regime, the models explained between 48.5% and 85.8% of the variation in FGP_30_. The best fit was for KCl (R^2^ = 0.858), followed by NaCl (R^2^ = 0.839), CaCl_2_ (R^2^ = 0.764), and MgCl_2_ (R^2^ = 0.754), whilst the mixture had the lowest coefficient of determination (R^2^ = 0.485). The slopes indicated estimated reductions in FGP_30_ of 0.27, 0.26, 0.26, 0.24, and 0.20 percentage points mM^−1^ for MgCl_2_, CaCl_2_, NaCl, KCl, and the mixture, respectively. Under continuous darkness, the models explained between 62.2% and 79.8% of the variation in FGP_30_. The best fit was obtained by MgCl_2_ (R^2^ = 0.798), followed by CaCl_2_ (R^2^ = 0.780), KCl (R^2^ = 0.757), NaCl (R^2^ = 0.715), and the mixture (R^2^ = 0.622). The slopes corresponded to estimated reductions of 0.27 percentage points mM^−1^ for the mixture, 0.26 for MgCl_2_ and CaCl_2_, 0.22 for KCl, and 0.21 for NaCl. For most of the individual salts, the slopes were slightly more negative under the light–dark regime than under continuous darkness, whereas CaCl_2_ exhibited similar slopes under both conditions and the mixture showed a more negative slope under continuous darkness.

Analysis of the kinetic parameters revealed differences between the light regimes even in the absence of salt. In the control seeds, germination was faster under continuous darkness, as indicated by the higher GRI and lower T_50_, which decreased from 5.09 days under the light–dark regime to 3.53 days under continuous darkness (Table 3). Germination was also more synchronised, as supported by the significantly higher Z and lower U under continuous darkness (Supplementary Table S3). This pattern was further supported by the significantly higher values for PV, GV_C*zabator*_, and CVG under continuous darkness, as well as by the higher values for MGT under the light–dark regime (Supplementary Table S3). Therefore, the light regime did not alter the final germination percentage under non-saline conditions, but it did affect its rate and temporal distribution. In the saline treatments, the kinetic response also depended on the concentration and type of salt. Overall, the mixture caused the most pronounced alteration in germination kinetics. Among the individual salts, KCl generally resulted in faster germination, whilst CaCl_2_ and MgCl_2_ caused the greatest delays, particularly at intermediate and high concentrations. Under the light–dark regime, the GRI decreased progressively as the salt concentration increased. With the mixture, it fell from 15.44% day^−1^ in the control to 3.03% day^−1^ at 22.5 mM and reached values close to or equal to zero at higher concentrations. At the same time, T_50_ increased, reaching its maximum values with CaCl_2_ and MgCl_2_ at 180 mM and with the mixture at 45 mM. The tendency towards faster germination under continuous darkness persisted in many salt treatments, although increasing salinity reduced GRI and increased T_50_, particularly with CaCl_2_, MgCl_2_, and the mixture (Table 3). The other kinetic indicators (PV, GV*_Czabator_*, GV*_Timson_*, CVG, and MGT) generally supported this pattern. Furthermore, variations in Z and U revealed changes in germination synchronisation and temporal dispersion depending on salt type, concentration, and light regime (Supplementary Table S3).

**Figure 5:**
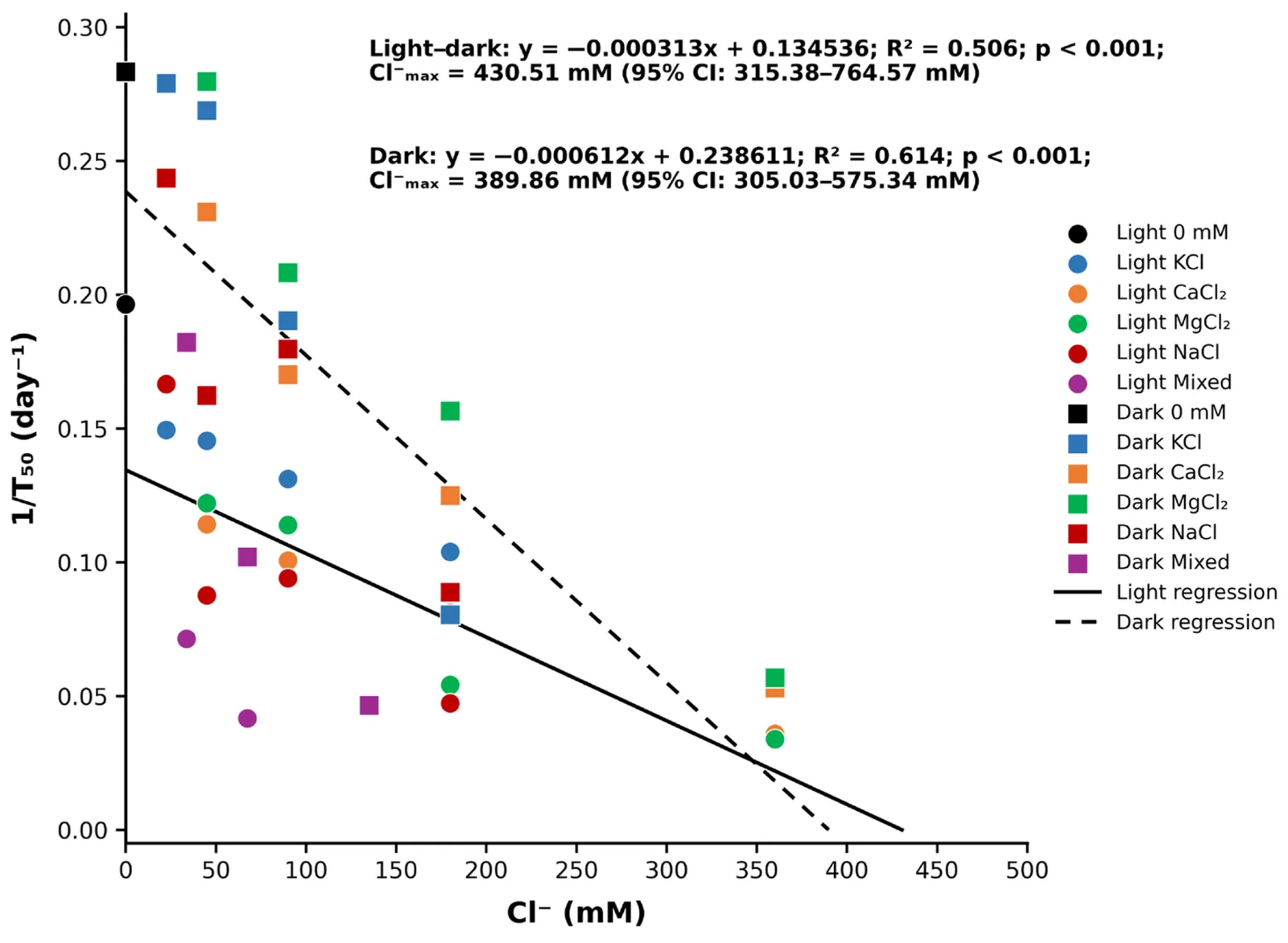
Relationship between the germination rate (1/T_50_, day^−1^) of *D. viscosa* seeds and increasing Cl^−^ concentration under the light–dark regime and continuous darkness during the 30-day salinity treatment. T_50_, time required to reach 50% of final germination.

To quantify the response of the germination rate to Cl^−^ concentration, the relationship between 1/T_50_ and the concentration of this ion was analysed under both light regimes (Figure 5). The relationship was negative and significant in both cases (*p* < 0.001). Under the light–dark regime, the model explained 50.6% of the variation in 1/T_50_ and yielded an estimated Cl^−^_max_ of 430.51 mM, whilst under continuous darkness it explained 61.4% of the variation and yielded an estimated Cl^−^_max_ of 389.86 mM. The slope was more negative under continuous darkness (*p* < 0.05). Furthermore, the salt type × light regime × Cl^−^ concentration interaction was significant (*p* < 0.05; Supplementary Table S4). The analyses carried out separately for each salt showed differences in significance and in the estimated thresholds (Table 4). Under the light–dark regime, the regressions were significant for KCl, CaCl_2_, and MgCl_2_ but not for NaCl or the salt mixture (*p* > 0.05). Under continuous darkness and in the pooled analysis of both light regimes, all regressions were significant; in the latter, CaCl_2_ and MgCl_2_ exhibited the highest values of Cl^−^_max_ (≈420 mM), followed by KCl and NaCl, whilst the mixture showed the lowest value (136.27 mM). Although the pooled regressions were significant, the wide 95% confidence intervals associated with some Cl^−^_max_ estimates indicate that their precision was limited. Therefore, the apparent sensitivity to Cl^−^ followed the order: mixture > NaCl > KCl > MgCl_2_ ≈ CaCl_2_. The slopes differed significantly between the salts under both light regimes (*p* < 0.001). The mixture exhibited the most negative slope under both the light–dark regime and continuous darkness. Under the light–dark regime, only NaCl and CaCl_2_ differed significantly, with NaCl exhibiting the more negative slope. KCl and MgCl_2_ did not differ significantly from either NaCl or CaCl_2_. Under continuous darkness, CaCl_2_ and MgCl_2_ exhibited the least negative slopes. When comparing the two regimes within each salt, only KCl showed a significant difference (*p* < 0.01) (Supplementary Table S4). These differences in slopes indicate that the relationship between 1/T_50_ and the Cl^−^ concentration varied among salt treatments, whilst the effect of the light regime depended on the salt type.

Following transfer to a salt-free medium, a high proportion of the seeds that had failed to germinate during salt exposure regained their germination capacity, although both the extent and rate of recovery depended on the treatment. High RP values were recorded with the individual salts, and TGP_45_ generally remained close to that of the control even after exposure to high concentrations, indicating that germination inhibition was largely reversible. At 360 mM, the TGP_45_ ranged from 64.70% to 84.10% under the light–dark regime and from 72.92% to 87.14% under continuous darkness. In contrast, the salt mixture substantially limited recovery. TGP_45_ decreased to 44.72% and 24.72% at 180 and 360 mM, respectively, under the light–dark regime, and to 58.63% and 16.73% in continuous darkness. Furthermore, the MGT during recovery generally ranged between 1 and 3 days with the individual salts, whilst with the mixture it increased to 7.62 and 7.06 days at 180 and 360 mM under the light–dark regime and to 3.64 and 5.29 days under continuous darkness, respectively (Table 5; Figure 1 and Figure 2). Therefore, at high concentrations, the mixture not only reduced total germination following recovery but also delayed the germination of seeds that retained their germination capacity.

### 2.2. Effect of Chloride Salts on Growth Parameters

Table 6 shows the results of the three-factor ANOVA for the effects of the light regime, salt type, salt concentration, and their interactions on the growth of *D. viscosa* seedlings. All main effects and interactions were significant for all growth parameters, except for the light regime × salt concentration interaction for total length. The analyses carried out separately for each light regime are presented in Supplementary Table S5.

In the control plates (0 mM), the light regime altered the distribution of growth. Under the light–dark regime, radicle growth predominated, with radicle and hypocotyl lengths of 33.11 and 2.82 mm, respectively, whilst under continuous darkness, hypocotyl elongation predominated, reaching 20.88 mm. Consequently, the radicle-to-hypocotyl ratio was significantly higher under the light–dark regime, although the total length and vigour index did not differ between the two regimes (*p* > 0.05; Table 7). Salinity reduced growth and the vigour index, with a response dependent on the salt type, concentration, and light regime. Under the light–dark regime, NaCl was the least limiting salt up to 90 mM and maintained a total length similar to the control up to this concentration, whilst CaCl_2_ did so up to 45 mM. KCl showed an intermediate response, whilst MgCl_2_ and the mixture were the most limiting treatments; the latter severely reduced the vigour index from the lowest concentrations. At 180 mM, growth was severely reduced with all individual salts, although KCl maintained the highest vigour index (Table 7). Under continuous darkness, the predominance of hypocotyl growth over radicle growth generally resulted in radicle-to-hypocotyl ratios lower than those observed under the light–dark regime. At 22.5 mM, KCl and CaCl_2_ maintained total lengths similar to the control, whilst radicle lengths did not differ significantly between the individual salts; KCl, CaCl_2_, and MgCl_2_ exhibited the highest vigour indices. Between 45 and 90 mM, NaCl and KCl generally maintained the greatest total lengths, and CaCl_2_ was also among the least restrictive treatments in terms of the radicle length and vigour index. MgCl_2_ and the mixture caused the greatest reductions. At 180 mM, NaCl was the least restrictive salt, though radicle growth was severely reduced with all individual salts. The mixture did not produce measurable seedlings at either 90 or 180 mM (Table 7). When growth variables and the vigour index were considered together, the salt mixture was the most inhibitory treatment while MgCl_2_ was, in general, the most restrictive individual salt. In contrast, NaCl best maintained growth under the light–dark regime up to 90 mM and in continuous darkness at high concentrations.

### 2.3. Correlation and Multivariate Analyses

Pearson correlations showed associations between the vigour index and germination parameters during the saline phase (Table 8). The vigour index correlated positively with FGP_30_ (r = 0.604) and GRI (r = 0.686), and negatively with T_50_ (r = −0.636) and RP (r = −0.643). These associations should be interpreted cautiously because of the mathematical dependence involving FGP_30_ and the conditional definition of RP. The correlations with GRI and T_50_ indicated that treatments with faster germination tended to exhibit higher vigour indices. The correlations showed the same trend under both light regimes, although under continuous darkness they were more pronounced for GRI (r = 0.788) and T_50_ (r = −0.739).

**Figure 6:**
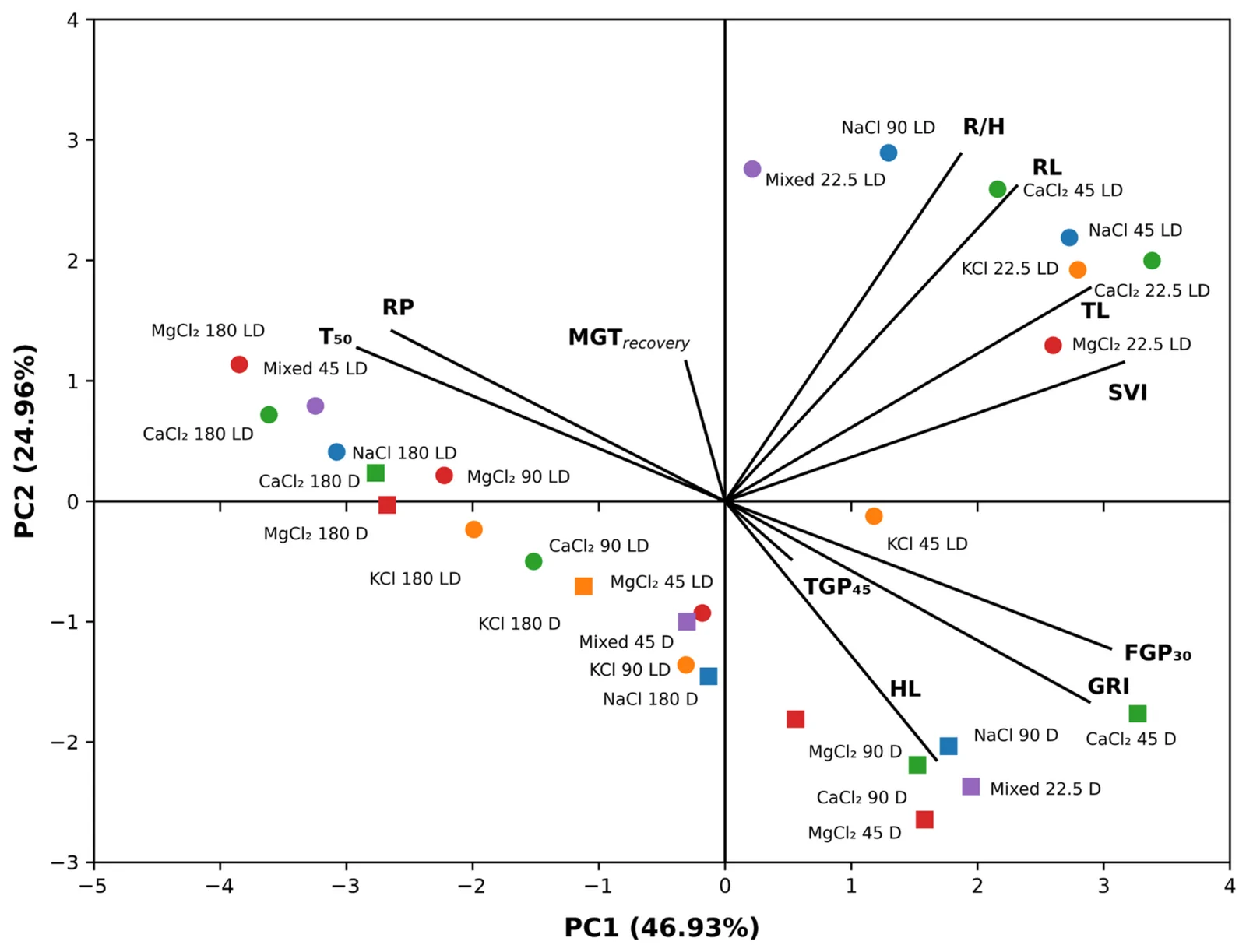
Principal component analysis biplot of germination, recovery, and seedling growth parameters of *D. viscosa*. Each point represents the mean value for the combination of salt type, concentration, and light regime. Circles correspond to the light–dark (LD) regime and squares to continuous darkness (D); colours identify NaCl (blue), KCl (orange), CaCl_2_ (green), MgCl_2_ (red), and the salt mixture (purple). Vectors represent the direction and magnitude of the variable loadings. FGP_30_, final germination percentage at 30 days; GRI, germination rate index; T_50_, time required to reach 50% of final germination; TGP_45_, total germination percentage after 30 days of salt exposure and 15 days of recovery; RP, recovery percentage; MGT*_recovery_*, mean germination time during recovery; RL, radicle length; HL, hypocotyl length; TL, total length; R/H, radicle-to-hypocotyl ratio; SVI, seedling vigour index.

Principal component analysis (PCA) enabled an examination of the relationships between germination, recovery, and seedling growth parameters. The first three components had eigenvalues greater than one and together accounted for 82.70% of the total variance. The first two components, shown in the biplot, together explained 71.89% of the total variance, with 46.93% attributed to PC1 and 24.96% to PC2 (Figure 6; Supplementary Table S6). PC1 showed positive loadings for FGP_30_, GRI, total length, and the seedling vigour index, and negative loadings for T_50_ and RP. This component separated treatments characterised by higher and faster germination and greater seedling growth from those associated with a longer germination delay and greater recovery following the removal of salt stress. The least restrictive treatments, generally corresponding to low or intermediate concentrations, were located on the positive side of PC1, whilst the most restrictive treatments shifted towards negative values. PC2 was primarily related to seedling architecture, with positive loadings for radicle length and the radicle-to-hypocotyl ratio, and a negative loading for hypocotyl length. This axis distinguished treatments associated with greater relative radicle development from those characterised by greater hypocotyl elongation. This separation was linked to the light regime; treatments under continuous darkness tended to be associated with hypocotyl elongation, whilst the light–dark regime was associated with greater relative radicle development. PC3 explained a further 10.80% of the variance and was dominated by TGP_45_ (Supplementary Table S6). Complementary PCAs showed an organisation consistent with this pattern. FGP_30_ and GRI loaded in the opposite direction to T_50_ and RP, whilst TGP_45_ and MGT*_recovery_* defined additional components. In turn, the growth variables differentiated radicle and total growth from hypocotyl elongation (Supplementary Table S6). Taken together, the correlations and PCAs showed that higher seedling vigour was associated with higher and faster germination during salt exposure, whereas the recovery variables represented a partially distinct component of the response.

## 3. Discussion

Soil salinisation in the Mediterranean region is on the rise as a result of reduced and erratic rainfall, as well as increased evapotranspiration and seawater intrusion—phenomena that may be exacerbated by climate change and human activity [1,4,5]. This growing accumulation of salts can limit seed germination, which is essential for recruitment, occupation of ecological niches, and distribution of species [30,31,32]. The sensitivity of germination to salinity varies among species and according to environmental conditions. Even among halophytes, the maximum NaCl concentration compatible with germination varies widely, with values ranging from approximately 0.26 to 1.7 M [10,33]. However, in halophytes, the highest germination percentages are generally achieved in the absence of salt stress [34] and, in natural environments, emergence tends to occur following periods of heavy rainfall, when dilution and leaching of salts temporarily reduce soil salinity [10]. In Mediterranean environments affected by salinity, early establishment also depends on the interaction between temperature, water availability and salt concentration [10,35]. The inhibitory effect of salinity results from a combination of osmotic and ionic components. The reduction in the water potential of the environment limits water uptake by the seed and delays imbibition, whilst excessive accumulation of ions can disrupt enzymatic activity, protein synthesis, respiration, and nutritional balance [6,36]. Furthermore, salt stress can promote an excessive accumulation of reactive oxygen species; an imbalance in these species leads to the oxidation of proteins, lipids, and nucleic acids, and affects viability and the progress of germination [37]. The germination response is also regulated by the balance between abscisic acid (ABA) and gibberellins (GAs). ABA promotes the maintenance of dormancy and inhibits germination, whilst GAs promote the breaking of dormancy and the emergence of the radicle; salinity can shift this balance towards reduced germination [32,38,39]. The extent of these alterations depends on the species, the composition and concentration of the salts, and other environmental factors; consequently, the germination response under saline conditions largely reflects the adaptive strategies developed by plants to establish themselves in habitats subject to water and salt stress [10,40].

In this context, the seeds of *Dittrichia viscosa* achieved similar final germination percentages under a light–dark regime and in continuous darkness in the absence of salinity (Figure 3). This result indicates that light was not necessary to complete germination and suggests that the seeds can be considered essentially non-photoblastic with respect to final germination percentage. However, continuous darkness increased the germination percentage during the early stages and reduced the time required to reach 50% of final germination; thus, the light regime influenced the kinetics, but not the final germination capacity. These results contrast with those of a previous study, which observed very low germination in continuous darkness and a marked requirement for light in *D. viscosa* [41]. Conversely, another study indicated that the absence of light may promote the breaking of dormancy in this species [25]. The differences between studies could be related to seed provenance, the environmental conditions experienced by the parent plants, the physiological state of the seed lots, and the storage and incubation conditions—factors that can modify dormancy and the germination response to environmental stimuli [42].

Under saline conditions, the germination response of *D. viscosa* depended on the light regime, type of salt, and concentration. The influence of the light regime was particularly evident at 90 and 180 mM (Figure 3; Table 3). At these concentrations, continuous darkness generally resulted in higher final germination percentages and germination rates than those recorded under the light–dark regime. It has also been observed that the response to salinity varies with light conditions in *Cistus monspeliensis*, a species in which the type of salt and concentration influenced germination and seedling growth [7]. Specifically, at the highest concentration tested in that species, continuous darkness exacerbated the reduction in germination caused by CaCl_2_ and the salt mixture [7]. Similarly, in *Suaeda salsa*, it has been documented that the germination response to salinity depends on light conditions and seed characteristics [43]. The influence of light under saline conditions does not, therefore, follow a consistent direction but rather depends on the species, concentration, and salt composition of the medium.

In addition to the light regime, the type of salt markedly altered the germination and subsequent growth of *D. viscosa*. These differences may be linked to the identity of the cation and to the osmotic and physicochemical properties of the solutions [44,45]. Generally speaking, KCl and NaCl were the least inhibitory salts during the germination phase of *D. viscosa*, although their relative rankings depended on the concentration and light regime. In contrast, CaCl_2_ and MgCl_2_ caused more pronounced reductions in the germination rate. The lower inhibition caused by KCl could be related to the role of K^+^ in osmotic adjustment and enzyme activation. However, even an essential nutrient can be harmful when its concentration exceeds the limits compatible with cellular homeostasis [46,47,48]. Furthermore, it has been shown that the presence of Ca^2+^ can modify the toxicity caused by sodium, potassium, and magnesium chlorides, confirming that the effects of the salts are not necessarily independent [45]. The differential response to salts partially coincides with that described in *C. monspeliensis* [7]. In this species, KCl and MgCl_2_ produced the greatest reductions in the final germination percentage at 120 mM. CaCl_2_ particularly affected germination kinetics, slowing the rate, increasing T_50_ and producing the lowest synchronisation at 80 mM. MgCl_2_ was also one of the salts most detrimental to seedling growth [7]. Therefore, both species showed high levels of inhibition caused by MgCl_2_, but differed in their response to KCl, which was comparatively less inhibitory in *D. viscosa*. The two species also exhibited different responses to the salt mixture. In *C. monspeliensis*, under the light–dark regime, the salt mixture was less inhibitory than KCl and MgCl_2_ at the highest concentration. Furthermore, NaCl and the salt mixture maintained the germination rate and promoted synchronisation, whilst the salt mixture proved less detrimental to initial growth [7]. In *D. viscosa*, by contrast, the mixture completely inhibited germination at concentrations of 90 mM and above under the light–dark regime and at 180 mM and above under continuous darkness. Different responses to NaCl and mixed saline media have also been documented in other species. In *S. salsa*, it was observed that NaCl and a combination of soil salts produced different effects on germination, and that the presence of Mg^2+^ and Ca^2+^ could modify the response to high concentrations of NaCl [49]. Taken together, the results obtained for *D. viscosa* indicate that the germination response varied with the composition and ionic ratios of the medium. These contrasting responses to mixed salts reinforce the conclusion that results obtained with individual salts cannot, by themselves, predict germination under more complex saline conditions. Although the equimolar mixture used here does not reproduce the composition of a specific saline soil, it incorporates the simultaneous presence of the major chloride salts and therefore provides a more ecologically relevant representation of ionic complexity than individual-salt treatments [7,40]. Salt stress in the present experiment involves both osmotic and ionic components. The osmotic component is associated with the reduction in solution water potential, which limits water uptake during imbibition and may delay germination. The ionic component is associated with the specific composition of the saline solutions, as the ions supplied may affect ionic homeostasis, nutrient balance, membrane stability and metabolic activity [6,7].

With regard to NaCl, the pattern observed is broadly consistent with previous studies on *D. viscosa*. A progressive reduction and delay in germination have been reported with increasing concentrations up to 171 mM [28], as well as particularly severe inhibition above 300 mM and virtually no germination at 400 mM [26]. In the present study, the response to NaCl was influenced by the light regime; *D. viscosa* maintained a germination percentage of 45.42% at 90 mM under the light–dark regime, and even 68.56% at 180 mM in continuous darkness. In *Tagetes erecta*, the germination percentage remained above 50% only up to 40 mM NaCl [50]. The response also varied widely among the other five Asteraceae species assessed. At 120 mM, the most marked reductions were recorded in *Gazania splendens*, *Gaillardia aristata*, and *Coreopsis grandiflora*, whose final percentages were 6.25, 6.25, and 2.50%, respectively. In *Ageratum houstonianum*, the maximum germination percentage was reached at 80 mM and was significantly higher than the control, whilst *Rudbeckia hirta* maintained percentages statistically similar to the control up to 80 mM and showed the highest relative tolerance among the species assessed [51]. Under the corresponding experimental conditions, the percentage maintained by *D. viscosa* at 180 mM under continuous darkness was higher than that recorded at 120 mM for any of those five species. Under the light–dark regime, however, its response at 90 mM was similar to those of *A. houstonianum* and *R. hirta* at 120 mM. These results indicate that the tolerance of *D. viscosa* to NaCl was comparatively high under continuous darkness but more moderate under the light–dark regime. In a comparison of three species from the tribe Inuleae, the germination capacity of *D. viscosa* was lower than that of *Limbarda crithmoides*, a halophyte characteristic of saline environments, but higher than that of *Inula helenium*, which is associated with non-saline environments [26]. This intermediate position is consistent with another comparative study of Asteraceae, in which *Aster tripolium* and *L. crithmoides* germinated at salt concentrations of up to 600 mM, whilst two species of *Dittrichia* failed to germinate under high-salinity conditions [52]. Overall, these comparisons suggest that, under the conditions tested, *D. viscosa* does not possess the germination capacity of the most specialised halophytes but can tolerate NaCl concentrations higher than those tolerated by some non-halophytic Asteraceae, particularly in continuous darkness.

Increased salinity reduced the germination rate index (GRI) and prolonged the time required to reach 50% of final germination (T_50_) (Table 3). The extent of these changes depended on the type of salt and light regime. At 180 mM, GRI decreased by between 82.9 and 99.7% under the light–dark regime and by between 69.2 and 93.2% under continuous darkness, considering the four individual salts, whilst T_50_ increased by between 89.0% and 479.6% under the light–dark regime and by between 218.7% and 434.8% under continuous darkness. These results show that germination rate may be more sensitive to salinity than the final germination percentage, particularly under continuous darkness. A similar pattern was observed in *Suaeda fruticosa* and *Limonium stocksii*, in which the Timson index decreased proportionally more than the final germination percentage [53]. In *Salicornia europaea*, the GRI also decreased with salinity, whilst T_50_ and MGT remained virtually unchanged [18]. This response contrasts with that of *D. viscosa*, in which T_50_ increased markedly before germination was virtually inhibited. In *Amaranthus albus* and *A. hybridus*, the MGT increased significantly from 150 mM NaCl onwards [33]. These comparisons highlight that the final percentage, rate, and time of germination provide complementary information on the response to salinity.

The negative relationship between the germination rate, expressed as 1/T_50_, and the Cl^−^ concentration showed that increasing the chloride concentration in the treatments progressively slowed the germination of *D. viscosa* (Figure 5; Supplementary Table S4). Although continuous darkness promoted faster germination in many treatments, the germination rate decreased more sharply with increasing Cl^−^ under continuous darkness in the combined analysis. However, the interactions between salt type, light regime, and Cl^−^ concentration, together with the differences observed in the slopes, indicate that this effect depended on the ionic composition of the treatment and not exclusively on the light regime. Among the significant regressions, the estimated Cl^−^_max_ values were generally higher for CaCl_2_ and MgCl_2_ and lower for the mixture. However, these estimates should be interpreted cautiously, as some regressions were based on a limited number of concentration means and had wide confidence intervals. Cl^−^_max_ was not estimated for the non-significant NaCl and mixture regressions under the light–dark regime (Table 4). The high Cl^−^_max_ values estimated for CaCl_2_ and MgCl_2_ do not contradict their strong effect on the GRI, as both parameters describe different aspects of germination dynamics and the model takes into account the Cl^−^ concentration, which in these salts is double the nominal molar concentration of the treatment. In *C. monspeliensis*, a Cl^−^_max_ of 491.2 mM was estimated from different chloride salts under the light–dark regime [7]. The values obtained for *D. viscosa* were lower. In the halophyte *S. maritima*, a Cl^−^_max_ of 1381 mM and a Na^+^_max_ of 1262 mM were estimated during germination in artificial seawater and in solutions of its main salts [40]. The proximity of these two thresholds indicated that Cl^−^ could limit the germination rate in a similar way to Na^+^ in mixed saline media. Furthermore, the combination of salts in the artificial seawater produced greater inhibition than the individual salts at the concentrations present in the mixture. In that study, germination in artificial seawater was higher than that observed in iso-osmotic PEG solutions, suggesting that osmotic restriction alone did not account for the response and that ion toxicity was an important constraint [40]. The lower values of Cl^−^_max_ obtained for *D. viscosa*, particularly with the mixture, are consistent with greater sensitivity of its germination rate to increases in Cl^−^. The lower threshold of the mixture was consistent with its strong inhibitory effect on FGP_30_, recovery and seedling growth. Cl^−^_max_ should be interpreted as a kinetic descriptor derived from the model and not as an absolute physiological limit for survival, final germination or establishment. This approach, based on the relationship between 1/T_50_ and Cl^−^ concentration, allows the response of the germination rate to be distinguished from that of the final germination percentage [7,35,40]. Furthermore, the statistical relationship with Cl^−^ does not demonstrate toxicity caused exclusively by the anion, as its concentration covaried with total salinity, osmolarity, ionic strength and the identity of the associated cation, although its excessive accumulation may contribute to metabolic and membrane alterations [46,48].

The behaviour during recovery altered the interpretation of the tolerance of *D. viscosa*. The high germination percentages achieved after removal of the individual salts indicate that the inhibition during exposure was primarily temporary and reversible, consistent with the maintenance of viable seeds in a state of quiescence (Table 5; Figure 1 and Figure 2). This response is consistent with previous observations in *D. viscosa* [26] and with the recovery described in *S. fruticosa*, L. *stocksii*, several Mediterranean species of *Limonium*, species of *Arthrocnemum*, *Lycium humile* and other halophytes from south-eastern Spain [12,53,54,55,56]. In contrast, some more sensitive glycophytes showed lower recoveries following the removal of salt stress [55,56,57,58]. Taken together, these studies show that the absence of germination during salt exposure does not necessarily represent an irreversible loss of germination capacity. This ability to postpone germination until salinity decreases is a common response in halophytes and tolerant species exposed to rainfall pulses or the temporary dilution of soil salts [10,59]. The salt mixture deviated from this pattern of reversibility, as its inhibitory effect persisted after the salts were removed and affected both the extent and rate of recovery (Table 5; Figure 1 and Figure 2). This effect could be related to ionic and oxidative alterations, given that oxidative stress has been associated with the response of *D. viscosa* to salinity [26]. Incomplete recovery could be due to the loss of viability in some of the seeds, persistent physiological inhibition, the induction of secondary dormancy, or a combination of these processes. In seeds of *A. macrostachyum* and *A. indicum*, differences in recovery were associated with the accumulation of H_2_O_2_ and malondialdehyde and with variations in the activity of antioxidant enzymes [60]. In other species, seeds have also been observed to remain in quiescence alongside losses in viability in the most concentrated treatments [12], whilst incomplete recovery has been linked to increased dormancy and some mortality [29]. However, these mechanisms cannot be distinguished on the basis of germination recovery alone and would require additional assessments of viability, dormancy, and oxidative stress.

Seedling growth showed that tolerance during germination did not necessarily predict the post-germination response of *D. viscosa*. Under the light–dark regime, NaCl maintained total length close to that of the control up to 90 mM, although it reduced the germination percentage. In contrast, KCl at 90 mM and MgCl_2_ at 45 mM allowed for relatively high germination percentages but reduced seedling elongation (Figure 3; Table 7). These contrasts indicate that the final germination percentage, total length, and seedling vigour index represent complementary components of the response to salinity and must be interpreted together to assess early establishment potential. This dissociation between germination and early growth has also been described in *S. salsa*, *Elymus farctus*, *A. albus*, *A. hybridus*, *C. monspeliensis*, and *Aegilops tauschii* [7,33,43,61,62]. Although both stages are affected by the osmotic and ionic components of salt stress, their physiological requirements differ. Germination depends on imbibition, seed-reserve mobilisation, and metabolic reactivation, whereas seedling growth requires the sustained maintenance of turgor, cell expansion and ionic homeostasis in meristematic tissues [6,48,61]. Consistent with this distinction, cotton exposed to mixed saline–alkali stress showed concurrent reductions in germination and root traits as stress increased. In that study, greater tolerance among cotton genotypes was associated with water uptake, adjustments in root morphological traits and changes in seed storage substances [63]. In the absence of salinity, the light regime altered the distribution of growth, but not total length or the vigour index. Under the light–dark regime, radicle development predominated, whilst continuous darkness favoured hypocotyl elongation (Table 7). In the presence of salinity, the salt mixture was the most restrictive treatment, while MgCl_2_ was, in general, the salt that caused the greatest reduction in vigour. In contrast, NaCl best maintained post-germination growth, whilst KCl showed an intermediate response. The high toxicity of Mg^2+^ to germination and radicle survival has also been observed in *Kalidium caspicum* [44]. The relative sensitivity of the radicle and hypocotyl varied with the salt and light regime, as reflected by changes in the radicle-to-hypocotyl ratio. This inter-organ variability has also been described in *E. farctus* and *Xanthoceras sorbifolium* [61,64]. Differences between salts may reflect the combined action of their osmotic properties and ionic composition, although the lower inhibition under NaCl may be consistent with a greater capacity to maintain ionic homeostasis [48]. Consequently, the final germination percentage, when considered in isolation, may overestimate the species’ establishment potential under the most restrictive saline treatments.

Pearson correlations and principal component analysis showed a convergent pattern and allowed PC1 to be interpreted as a response gradient during salt exposure, which integrated germination, its temporal dynamics and seedling vigour (Table 8; Figure 6; Supplementary Table S6). The associations of GRI and T_50_ with the vigour index suggest that the rate and delay of germination were related to post-germination growth. In a comparative analysis of *Bidens pilosa*, *Centaurea cyanus*, *Echinacea purpurea*, *Limonium sinuatum*, *Lobularia maritima*, and *Oenothera biennis*, a longer germination time was associated with a greater reduction in growth and lower vigour under water stress [65]. In *S. europaea*, by contrast, T_50_ and MGT contributed very little to the principal component related to salinity [18]. Similarly, in *A. albus* and *A. hybridus*, multivariate analysis enabled the identification of the growth variables most closely related to saline conditions and distinguished them from those less suitable as indicators of tolerance [33]. The multivariate behaviour of the recovery variables in *D. viscosa* indicated that the germination capacity retained after salt removal represents a component that is partially distinct from the vigour developed during salt exposure. PC2 primarily represented the distribution of growth between the radicle and the hypocotyl and its relationship with the light regime. The covariation between the morphological variables of the seedlings, and their differentiation from other components of germination has also been observed in the six ornamental species mentioned above [65]. In cultivated C_3_ and C_4_ species, discriminant analysis likewise showed that the variables most affected by salinity varied between germination, seedling growth, and subsequent stages of development [48].

Overall, the present results suggest that *D. viscosa* could be considered in future evaluations for the revegetation of degraded or moderately salinised environments. Although the species readily colonises disturbed substrates and has been proposed for phytoremediation applications [66], it may behave as a native-invasive species in Mediterranean salt marshes with low or moderate salinity, where it could compete with halophytic communities of conservation interest [67]. Consequently, any practical application should be preceded by a site-specific ecological assessment.

This study provides a controlled assessment of the germination response and early growth of *D. viscosa* seedlings using seeds from a single population. The agar-based experimental system allowed the effects of salinity treatments and light regimes to be examined under reproducible conditions, whilst growth assessment was carried out at a single early stage of development. Due to its experimental scope, the study did not include subsequent validation in soil substrates or under field conditions. Given that seed responses may vary between populations and that natural saline soils exhibit greater physical, chemical and biological complexity and are subject to fluctuating temperatures, future trials involving additional populations, soil substrates, and field conditions would be valuable in broadening the ecological and applied relevance of these results.

## 4. Materials and Methods

### 4.1. Study Species, Seed Collection and Habitat

In October 2022, numerous mature seed heads of *Dittrichia viscosa* were randomly collected from 30 individuals distributed throughout a natural population located in Torrequebradilla in the province of Jaén, Spain (37.91° N, 3.67° W; 360 m a.s.l.). In addition, voucher specimens were collected from three individuals in the population and deposited in the Herbarium of the Centre for Scientific Collections at the University of Almería under the accession numbers HUAL 30825–30827 [68]. The area has a Mediterranean pluviseasonal oceanic bioclimate and is situated within the dry meso-Mediterranean bioclimatic zone [69]. During the period 1985–2014, the average annual temperature was 16.5 °C and the average annual rainfall was 421.4 mm according to the Climate Map of Andalusia [70]. The population occurred on disturbed soils developed from detrital-carbonate materials consisting of siliceous sands interbedded with marls [71]. At this site, *D. viscosa* formed dense stands in association with ruderal and salt-tolerant species, including *Piptatherum miliaceum* (L.) Coss., *Lygeum spartum* L., *Atriplex halimus* L., *A. prostrata* DC., *Sonchus tenerrimus* L., *Hordeum marinum* Huds., *Frankenia pulverulenta* L., and *Spergularia marina* (L.) Besser.

After collection, the achenes were manually separated from the capitula and other plant debris, and the pappus was removed. The achenes obtained from all sampled individuals were pooled to form a single seed lot. They were then examined under a stereomicroscope, and those that were unfilled, immature, or visibly damaged were discarded. Only mature achenes showing no apparent external damage were selected for the experiments. Hereafter, for the sake of simplicity, the term seeds is used to refer to the achenes used in the germination trials. The selected seeds were stored in the dark at room temperature from the time of harvest in October 2022 to the start of the germination trials in January 2023.

### 4.2. Experimental Design, Germination and Recovery Assays

The germination assay was carried out in plastic Petri dishes 9 cm in diameter containing 0.6% (*w*/*v*) Plant Agar, to which the corresponding salts were added in the saline treatments [26]. The culture media were prepared by dissolving the appropriate amount of each salt in distilled water, according to the concentration of each treatment, and then adding Plant Agar until a final concentration of 0.6% (*w*/*v*) was reached. No salts were added to the control treatment. Each medium was heated until the agar had completely melted and was dispensed into tubes in 20 mL aliquots. The tubes were autoclaved at 121 °C for 20 min and, under sterile conditions, the contents of each tube were subsequently poured into a Petri dish. A factorial design with three factors was used: salt type, salt concentration, and light regime. Four chloride salts (NaCl, KCl, CaCl_2_, and MgCl_2_) and a mixture of the four salts in a molar ratio of 1:1:1:1 were evaluated by following a previously described approach [7]. The nominal concentration of the mixture corresponded to the total molar concentration, distributed equally amongst its four components. The physicochemical characteristics of the prepared salt solutions, including chloride concentration, ionic strength, electrical conductivity, pH, and estimated osmotic potential, are presented in Table 9.

For each individual salt and their mixture, six concentrations were tested: 0 (control), 22.5, 45, 90, 180, and 360 mM. The 0 mM control, consisting of the same culture medium without any added salts, was included in each salt series under both light regimes as a common reference [7]. Each treatment was incubated under two light regimes: 12 h of light and 12 h of darkness, with a photosynthetic photon flux density of 100 μmol photons m^−2^ s^−1^ provided by daylight fluorescent tubes (light–dark regime); and continuous darkness. For each treatment, four replicates of 25 seeds were used, with each Petri dish considered an experimental unit. The 0 mM control was replicated within each of the five experimental series, resulting in 20 chemically identical control dishes per light regime. In total, the experiment comprised 240 Petri dishes (5 salt-treatment series × 6 concentrations × 2 light regimes × 4 replicates).

Prior to sowing, the seeds were surface-sterilised by immersion in a 5% sodium hypochlorite solution for 5 min and subsequently rinsed with sterile distilled water [7]. After sowing, the plates were sealed with Parafilm® M (Bemis Company, Inc., Neenah, WI, USA) to minimise moisture loss and incubated in a germination chamber under a thermoperiod of 12 h at 20 °C and 12 h at 10 °C. Under the light–dark regime, the 12 h at 20 °C corresponded to the light period, whilst the 12 h at 10 °C corresponded to the dark period. Plates assigned to continuous darkness were kept wrapped in aluminium foil throughout the experiment and subjected to the same thermoperiod. The plates were randomly distributed within each germination chamber and were randomly rearranged at each assessment date to minimise potential positional effects. Germination was recorded every 24 h for 30 days. Germination counts in the plates incubated in continuous darkness were carried out under a low-intensity green safety light [72,73]. A seed was considered to have germinated when the radicle had emerged at least 0.5 mm through the pericarp [74,75]. After 30 days of exposure to the saline treatments, seeds that had not germinated were removed from the saline media, rinsed with sterile distilled water, and transferred to new Petri dishes containing 0.6% (*w*/*v*) Plant Agar without added salts. The plates were maintained for 15 days under the same temperature and light conditions as those used in the initial experiment. The viability of seeds that remained ungerminated after the salt treatment and recovery period was assessed using the tetrazolium test according to ISTA criteria [76]. The initial viability of the seed lot was 84%. Germination percentages during salt exposure and total germination after recovery were calculated relative to the total number of viable seeds in each replicate.

### 4.3. Germination and Recovery Parameters

Based on the daily counts, various parameters were calculated to characterise the percentage and rate of germination, their temporal distribution and synchrony, and the seeds’ ability to recover following the removal of salt stress [12,17,18]. The equations used, their biological interpretation, and the corresponding references are presented in Table 10.

### 4.4. Seedling Analysis

Ten days after germination, the hypocotyl length (HL) and radicle length (RL) of the seedlings were measured [18]. From these values, the mean total length of the seedlings (TL = HL + RL) and the radicle-to-hypocotyl length ratio (R/H = RL/HL) were calculated. The seedling vigour index (SVI) was calculated as SVI = FGP_30_ × TL, where FGP_30_ is the final germination percentage at 30 days and TL is the mean total seedling length expressed in centimetres [7,84].

### 4.5. Statistical Analysis

One-way ANOVAs were carried out to assess, within each light regime, the effect of concentration for each type of salt and the differences between salts at each concentration. Two-factor ANOVAs (salt type × concentration) were used to determine, separately for each light regime, the effects of both factors and their interaction, whilst three-factor ANOVAs jointly assessed the effects of the light regime, salt type, concentration, and their interactions on germination, recovery, and seedling growth. Final germination proportions at 14, 21, and 30 days (FGP_14_, FGP_21_, and FGP_30_), as well as total germination after 30 d of salt treatment and 15 d of recovery (TGP_45_), were analysed separately using generalised linear models (GLMs) with a binomial error structure [85]. The models included light regime, salt treatment, and their interaction as fixed effects. The five chemically identical groups of 0 mM controls were treated as a single control level, resulting in 26 salt-treatment levels: one control and 25 combinations of salt types and non-zero concentrations. Pairwise comparisons were performed using Tukey-adjusted contrasts (*p* < 0.05). The values for RP were transformed using the arcsine of the square root of the quotient of the percentage value and 100. The hypocotyl length and radicle-to-hypocotyl ratio were transformed using the natural logarithm of the value increased by one unit, whilst radicle length and the vigour index were transformed using the square root of the original value. When a germination parameter could not be calculated because no seeds germinated, it was treated as undefined and excluded from the ANOVA for that specific parameter. For the ANOVA-based analyses, means were compared using Tukey’s test (*p* < 0.05). Linear regressions were fitted between salt concentration and FGP_30_ for each combination of salt and light regime. The germination rate, expressed as the inverse of T_50_, was related to the Cl^−^ concentration. Cl^−^_max_ was estimated as the concentration corresponding to a germination rate of zero, obtained by the intersection of the regression line with the concentration axis [7,40]. Pearson correlations were calculated between the germination and recovery parameters and the seedling growth parameters, both for the complete dataset and for each light regime. Principal component analysis (PCA) was performed using the standardised means for each combination of light regime, salt type, and concentration. The integrated PCA included only those treatments with complete data; in addition, separate PCAs were carried out for germination and recovery parameters and for post-germination growth parameters. All analyses were performed using Statgraphics Centurion XIX (version 19), utilising the University of Jaén’s institutional licence.

## 5. Conclusions

The seeds of *D. viscosa* germinated under both the light–dark regime and continuous darkness, although the absence of light accelerated germination and attenuated the inhibitory effect of several individual salts. The response varied with the salt concentration and salt type, indicating that the species’ tolerance cannot be inferred solely from its response to NaCl. The seeds retained considerable germination capacity under low and moderate concentrations of the individual salts and showed substantial germination recovery after the stress was removed, which could allow germination to be postponed until rainfall temporarily reduces soil salinity. The treatment with a salt mixture showed more intense and persistent inhibition, whilst seedling growth was more sensitive than germination and varied depending on the salt type, salt concentration, and light regime. The differences among the estimated Cl^−^_max_ values and the partial separation among germination during exposure, subsequent recovery and initial growth indicate that the salt tolerance of *D. viscosa* comprises related but not equivalent components. Taken together, these results are consistent with the behaviour of a halotolerant glycophyte capable of overcoming transient saline episodes but whose establishment may be limited by post-germination sensitivity and by more restrictive saline conditions. These characteristics justify assessing its potential use in the revegetation of degraded or moderately salinised soils.

## Figures and Tables

**Table 1:** Analysis of deviance of binomial generalised linear models examining the effects of light regime, saline treatment, and their interaction on final germination percentage at 14, 21, and 30 days (FGP_14_, FGP_21_, and FGP_30_) and total germination after 30 days of salt treatment plus 15 days of recovery (TGP_45_) in *D. viscosa*.

Parameter	Light Regime (L)		Saline Treatment (T)		L × T	
	d.f.	Deviance	d.f.	Deviance	d.f.	Deviance
FGP_14_	1	60.35 ***	25	2196.76 ***	25	90.24 ***
FGP_21_	1	40.42 ***	25	2291.46 ***	25	91.48 ***
FGP_30_	1	35.29 ***	25	2284.69 ***	25	69.49 ***
TGP_45_	1	0.97 ns	25	389.94 ***	25	20.09 ns

d.f., degrees of freedom; ns, not significant; ***, *p* < 0.001.

**Table 2:** Three-way ANOVA for the effects of light regime, salt type, salt concentration, and their interactions on seed germination and recovery parameters of *D. viscosa*. Abbreviations: PV, peak value; GV*_Timson_*, modified Timson index; GV*_Czabator_*, germination value; CVG, coefficient of velocity of germination; GRI, germination rate index; MGT, mean germination time; T_50_, time to 50% of final germination; Z, synchrony; U, uncertainty; RP, recovery percentage; MGT*_recovery_*, mean germination time during recovery.

Parameter	Light Regime (L)		Salt (S)		Concentration (C)		L × S		L × C		S × C		L × S × C	
	d.f.	F-Ratio	d.f.	F-Ratio	d.f.	F-Ratio	d.f.	F-Ratio	d.f.	F-Ratio	d.f.	F-Ratio	d.f.	F-Ratio
PV	1	226.32 ***	4	30.07 ***	5	315.81 ***	4	1.57 ns	5	18.14 ***	20	5.59 ***	20	1.25 ns
GV*_Timson_*	1	197.38 ***	4	86.85 ***	5	629.15 ***	4	0.37 ns	5	19.87 ***	20	17.06 ***	20	6.43 ***
GV*_Czabator_*	1	166.68 ***	4	21.64 ***	5	189.32 ***	4	1.65 ns	5	15.40 ***	20	6.69 ***	20	1.71 *
CVG	1	162.05 ***	4	21.64 ***	4	167.85 ***	4	1.72 ns	4	7.08 ***	16	11.54 ***	16	2.01 *
GRI	1	220.61 ***	4	38.14 ***	5	404.69 ***	4	1.10 ns	5	14.32 ***	20	7.39 ***	20	2.12 **
MGT	1	361.95 ***	4	90.23 ***	4	459.85 ***	4	12.57 ***	4	26.63 ***	16	115.43 ***	16	18.31 ***
T_50_	1	402.81 ***	4	114.67 ***	4	513.46 ***	4	17.20 ***	4	32.63 ***	16	125.95 ***	16	23.40 ***
Z	1	135.39 ***	4	4.02 **	4	47.94 ***	4	0.85 ns	4	10.37 ***	16	6.37 ***	16	1.93 *
U	1	27.01 ***	4	18.93 ***	4	64.60 ***	4	5.77 ***	4	97.35 ***	16	12.01 ***	16	23.37 ***
RP	1	400.47 ***	4	65.20 ***	4	239.84 ***	4	9.31 ***	4	36.07 ***	16	66.14 ***	16	20.67 ***
MGT*_recovery_*	1	92.68 ***	4	103.01 ***	4	86.47 ***	4	10.94 ***	4	3.15 *	16	28.25 ***	16	13.00 ***

d.f., degrees of freedom; ns, not significant; *, *p* < 0.05; **, *p* < 0.01; ***, *p* < 0.001.

**Table 3:** Germination responses of *D. viscosa* seeds to chloride salts under two light regimes. Abbreviations: GRI, germination rate index; T_50_, time to 50% of final germination.

Light Regime	Control	Salt	Concentration (mM)				
			22.5	45	90	180	360
			GRI (% day^−1^)				
Light–dark	15.44 ± 0.63 aB	NaCl	14.90 ± 1.56 aA	7.11 ± 0.81 bC	4.06 ± 0.47 bcB	0.65 ± 0.05 cB	0.00 ± 0.00 cA
		KCl	12.58 ± 1.39 abAB	11.46 ± 1.09 bA	9.65 ± 0.80 bA	2.64 ± 0.31 cA	0.00 ± 0.00 cA
		CaCl_2_	9.69 ± 0.71 bB	8.25 ± 0.81 bcAB	4.64 ± 0.67 cB	0.18 ± 0.03 dBC	0.00 ± 0.00 dA
		MgCl_2_	12.11 ± 0.92 bAB	9.60 ± 0.86 bAB	3.36 ± 0.43 cB	0.04 ± 0.02 cBC	0.00 ± 0.00 cA
		Mixed	3.03 ± 0.40 bC	0.38 ± 0.09 bcC	0.00 ± 0.00 cC	0.00 ± 0.00 cC	0.00 ± 0.00 cA
Dark	20.92 ± 1.46 aA	NaCl	15.43 ± 1.31 abB	14.64 ± 1.62 abB	11.85 ± 1.10 bcB	6.45 ± 0.74 cdA	0.00 ± 0.00 dA
		KCl	22.15 ± 1.55 aA	21.37 ± 1.42 aA	15.65 ± 1.00 aA	5.34 ± 0.54 bA	0.00 ± 0.00 bA
		CaCl_2_	18.51 ± 1.43 abAB	14.54 ± 1.41 abB	11.83 ± 0.95 bB	1.42 ± 0.16 cB	0.00 ± 0.00 cA
		MgCl_2_	23.31 ± 0.65 aA	18.10 ± 0.41 abAB	12.48 ± 0.58 bAB	1.43 ± 0.17 cB	0.00 ± 0.00 cA
		Mixed	15.68 ± 0.49 aB	6.35 ± 0.58 bC	0.05 ± 0.03 bC	0.00 ± 0.00 bB	0.00 ± 0.00 bA
			T_50_ (days)				
Light–dark	5.09 ± 0.40 cA	NaCl	6.00 ± 0.69 cB	11.40 ± 1.25 bB	10.62 ± 1.01 bB	21.12 ± 1.70 aB	–
		KCl	6.69 ± 0.74 bcB	6.88 ± 0.43 bcB	7.62 ± 0.38 abB	9.62 ± 0.66 aC	–
		CaCl_2_	8.75 ± 0.72 cB	9.94 ± 0.48 bcB	12.31 ± 0.34 bB	27.83 ± 1.20 aA	–
		MgCl_2_	8.19 ± 0.83 cdB	8.77 ± 0.34 cB	18.44 ± 2.07 bA	29.50 ± 0.00 aAB	–
		Mixed	14.00 ± 1.21 bA	24.00 ± 1.84 aA	–	–	–
Dark	3.53 ± 0.18 cB	NaCl	4.10 ± 0.31 bcB	6.16 ± 0.68 bB	5.56 ± 0.41 bC	11.25 ± 1.13 aC	–
		KCl	3.58 ± 0.12 bcB	3.72 ± 0.21 bcC	5.25 ± 0.06 bC	12.44 ± 1.22 aBC	–
		CaCl_2_	4.33 ± 0.31 cdAB	5.88 ± 0.57 bcBC	8.00 ± 0.49 bB	18.88 ± 1.42 aA	–
		MgCl_2_	3.58 ± 0.15 cB	4.80 ± 0.11 bcBC	6.39 ± 0.17 bC	17.58 ± 1.56 aAB	–
		Mixed	5.49 ± 0.52 cA	9.80 ± 0.77 bA	21.50 ± 0.00 aA	–	–

Values are means ± standard error (SE). Different lowercase letters indicate significant differences among concentrations within the same salt type and light regime according to Tukey’s HSD test (*p* < 0.05). Different uppercase letters indicate significant differences among salt types within the same concentration and light regime according to Tukey’s HSD test (*p* < 0.05). For the control, uppercase letters indicate significant differences between light regimes. A dash (–) indicates that the parameter was not defined.

**Table 4:** Chloride-based regression parameters for germination rate (1/T_50_) of *D. viscosa* seeds under the light–dark regime, continuous darkness, and pooled datasets.

Light Regime	Salt	n	Regression Equation (1/T_50_ = a + b [Cl^−^])	R^2^	*p*	Cl^−^_max_ (mM)	95% CI (mM)
Light–dark	NaCl	5	1/T_50_ = 0.169429 − 0.000755 [Cl^−^]	0.770	0.0506	NE	–
	KCl	5	1/T_50_ = 0.174021 − 0.000426 [Cl^−^]	0.807	0.0384	408.78	241.88–3455.60
	CaCl_2_	5	1/T_50_ = 0.155030 − 0.000365 [Cl^−^]	0.783	0.0459	424.29	255.19–8654.04
	MgCl_2_	5	1/T_50_ = 0.158442 − 0.000402 [Cl^−^]	0.800	0.0404	393.96	241.64–3296.61
	Mixture	3	1/T_50_ = 0.180576 − 0.002293 [Cl^−^]	0.888	0.2174	NE	–
Dark	NaCl	5	1/T_50_ = 0.257306 − 0.000973 [Cl^−^]	0.845	0.0271	264.45	169.81–993.70
	KCl	5	1/T_50_ = 0.301768 − 0.001205 [Cl^−^]	0.972	0.0020	250.36	203.64–336.21
	CaCl_2_	5	1/T_50_ = 0.254449 − 0.000607 [Cl^−^]	0.925	0.0090	419.24	307.08–746.66
	MgCl_2_	5	1/T_50_ = 0.285232 − 0.000654 [Cl^−^]	0.970	0.0022	436.25	354.25–588.67
	Mixture	4	1/T_50_ = 0.254044 − 0.001701 [Cl^−^]	0.908	0.0473	149.31	91.50–3184.35
Pooled light regimes	NaCl	10	1/T_50_ = 0.213368 − 0.000864 [Cl^−^]	0.592	0.0092	246.94	167.01–629.88
	KCl	10	1/T_50_ = 0.237895 − 0.000816 [Cl^−^]	0.551	0.0140	291.71	190.02–925.18
	CaCl_2_	10	1/T_50_ = 0.204740 − 0.000486 [Cl^−^]	0.669	0.0038	421.14	301.14–821.46
	MgCl_2_	10	1/T_50_ = 0.221837 − 0.000528 [Cl^−^]	0.614	0.0074	420.15	290.26–960.64
	Mixture	7	1/T_50_ = 0.204220 − 0.001499 [Cl^−^]	0.604	0.0398	136.27	83.81–1339.71

Pooled analyses combined the values obtained under the light–dark regime and continuous darkness for each salt. n represents the number of points included in each regression. T_50_, time required to reach 50% of final germination; Cl^−^_max_, estimated maximum chloride concentration at which germination rate reaches zero; CI, confidence interval; NE, not estimable.

**Table 5:** Germination recovery of *D. viscosa* seeds after exposure to chloride salts under two light regimes. Abbreviations: TGP_45_, total germination percentage after 30 d of salt treatment and 15 d of recovery; RP, recovery percentage; MGT*_recovery_*, mean germination time during recovery.

Light Regime	Control	Salt	Concentration (mM)				
			22.5	45	90	180	360
			TGP_45_ (%)				
Light–dark	82.11 ± 1.90 aA	NaCl	89.67 ± 4.09 aA	81.61 ± 6.38 aA	75.83 ± 4.38 aA	79.88 ± 8.17 aA	79.38 ± 6.80 aA
		KCl	87.95 ± 4.57 aA	93.56 ± 4.03 aA	85.38 ± 6.55 aA	79.90 ± 1.96 bA	64.70 ± 6.24 bA
		CaCl_2_	86.45 ± 7.06 aA	96.43 ± 3.57 aA	91.48 ± 5.90 aA	90.37 ± 3.67 aA	84.10 ± 5.35 aA
		MgCl_2_	91.40 ± 5.21 aA	93.51 ± 2.33 aA	80.52 ± 1.79 bA	79.20 ± 5.30 aA	83.87 ± 1.12 aA
		Mixed	87.01 ± 6.38 aA	95.58 ± 2.60 aA	85.62 ± 2.53 aA	44.72 ± 5.39 cB	24.72 ± 1.72 cB
Dark	82.16 ± 3.60 aA	NaCl	77.56 ± 6.34 aB	83.63 ± 7.90 aA	74.08 ± 6.05 aA	77.09 ± 3.40 aA	77.09 ± 7.63 aA
		KCl	90.99 ± 3.85 aA	89.85 ± 5.92 aA	88.46 ± 4.97 aA	81.87 ± 3.12 aA	74.11 ± 6.19 aA
		CaCl_2_	93.06 ± 4.17 aA	91.81 ± 4.84 aA	92.92 ± 4.10 aA	87.98 ± 0.99 aA	87.14 ± 5.62 aA
		MgCl_2_	94.53 ± 0.85 aA	96.30 ± 2.30 aA	85.97 ± 3.15 aA	89.37 ± 4.03 aA	72.92 ± 2.08 bA
		Mixed	90.64 ± 2.66 aA	97.78 ± 1.28 aA	92.94 ± 3.23 aA	58.63 ± 5.45 cB	16.73 ± 2.12 dB
			RP (%)				
Light–dark	–	NaCl	0.00 ± 0.00 dC	25.00 ± 2.04 cC	55.36 ± 5.31 bB	76.55 ± 6.16 aA	79.38 ± 6.80 aA
		KCl	25.00 ± 2.53 bB	55.56 ± 5.67 aB	50.00 ± 5.68 aB	70.83 ± 2.41 aA	64.70 ± 6.24 aA
		CaCl_2_	8.33 ± 0.62 bC	83.33 ± 8.50 aA	81.11 ± 8.20 aA	89.69 ± 3.90 aA	84.10 ± 5.35 aA
		MgCl_2_	33.33 ± 3.12 cB	38.89 ± 4.16 cBC	63.27 ± 6.17 bAB	78.98 ± 5.23 abA	83.87 ± 1.12 aA
		Mixed	79.91 ± 6.37 aA	95.42 ± 2.67 aA	85.62 ± 2.53 aA	44.72 ± 5.39 bB	24.72 ± 1.72 cB
Dark	–	NaCl	0.00 ± 0.00 cB	0.00 ± 0.00 cC	10.42 ± 1.05 bcBC	21.88 ± 1.94 bD	77.09 ± 8.40 aA
		KCl	0.00 ± 0.00 cB	0.00 ± 0.00 cC	0.00 ± 0.00 cD	38.33 ± 4.41 bC	74.11 ± 6.19 aA
		CaCl_2_	0.00 ± 0.00 cB	12.50 ± 2.50 bBC	6.25 ± 0.75 bcCD	84.00 ± 1.28 aA	87.14 ± 5.62 aA
		MgCl_2_	0.00 ± 0.00 dB	25.00 ± 2.04 cB	17.50 ± 1.44 cB	86.76 ± 3.55 aA	72.92 ± 2.08 bA
		Mixed	12.50 ± 1.44 cA	88.75 ± 6.57 aA	92.83 ± 3.26 aA	58.63 ± 5.45 bB	16.73 ± 2.12 cB
			MGT*_recovery_* (days)				
Light–dark	–	NaCl	–	1.00 ± 0.00 bB	1.38 ± 0.22 bC	1.58 ± 0.17 bB	2.65 ± 0.16 aB
		KCl	3.75 ± 0.31 aA	1.17 ± 0.10 cB	1.00 ± 0.00 cC	1.69 ± 0.24 bcB	2.45 ± 0.27 bB
		CaCl_2_	2.00 ± 0.20 bB	2.22 ± 0.28 abA	1.58 ± 0.06 bC	1.98 ± 0.15 bB	3.05 ± 0.28 aB
		MgCl_2_	2.00 ± 0.20 bB	3.00 ± 0.20 aA	2.50 ± 0.19 abB	2.51 ± 0.27 abB	3.14 ± 0.16 aB
		Mixed	1.87 ± 0.25 cB	2.26 ± 0.19 bA	3.33 ± 0.16 bA	7.62 ± 0.62 aA	7.06 ± 0.50 aA
Dark	–	NaCl	–	–	1.50 ± 0.20 aAB	1.42 ± 0.05 aB	1.83 ± 0.18 aB
		KCl	–	–	–	2.17 ± 0.51 aB	1.93 ± 0.24 aB
		CaCl_2_	–	2.00 ± 0.20 abA	1.00 ± 0.20 bB	1.95 ± 0.28 abB	2.57 ± 0.28 aB
		MgCl_2_	–	1.00 ± 0.20 cB	2.00 ± 0.20 bA	1.38 ± 0.10 bcB	2.92 ± 0.28 aB
		Mixed	1.50 ± 0.29 cA	1.53 ± 0.14 cAB	2.11 ± 0.19 cA	3.64 ± 0.27 bA	5.29 ± 0.49 aA

Values are means ± standard error (SE). For TGP_45_, different lowercase letters indicate significant differences among concentrations within the same salt type and light regime, whereas different uppercase letters indicate significant differences among salt types within the same concentration and light regime, based on Tukey-adjusted pairwise contrasts from binomial generalised linear models (*p* < 0.05). For RP and MGT recovery, lowercase and uppercase letters indicate significant differences according to Tukey’s HSD test (*p* < 0.05) for the same corresponding comparisons. For the control, uppercase letters indicate significant differences between light regimes. A dash (–) indicates that the parameter was not defined.

**Table 6:** Three-way ANOVA for the effects of the light regime, salt type, salt concentration, and their interactions on seedling growth parameters of *D. viscosa*.

Source of Variation	d.f.	Hypocotyl (H)	Radicle (R)	R/H	Total Length	Seedling Vigour Index
Light regime (L)	1	6265.61 ***	720.23 ***	6774.83 ***	8.46 **	231.80 ***
Salt (S)	4	107.87 ***	175.89 ***	97.75 ***	116.85 ***	211.95 ***
Concentration (C)	4	689.89 ***	1522.25 ***	876.93 ***	472.45 ***	1737.28 ***
L × S	4	28.66 ***	33.78 ***	74.83 ***	19.18 ***	15.03 ***
L × C	4	184.59 ***	80.27 ***	479.97 ***	1.55 ns	39.02 ***
S × C	16	24.89 ***	92.00 ***	81.07 ***	35.09 ***	78.07 ***
L × S × C	16	19.37 ***	25.00 ***	55.97 ***	9.93 ***	16.76 ***

The 360 mM treatment was excluded from the analysis because no measurable seedlings were obtained. Abbreviations: d.f., degrees of freedom; H, hypocotyl length; R, radicle length; R/H, radicle-to-hypocotyl ratio; ns, not significant; **, *p* < 0.01; ***, *p* < 0.001.

**Table 7:** Seedling growth parameters of *D. viscosa* under different light regimes, chloride salts, and salt concentrations.

Light Regime	Control	Salt	Concentration (mM)			
			22.5	45	90	180
			Hypocotyl Length (mm)			
Light–dark	2.82 ± 0.06 aB	NaCl	2.99 ± 0.01 aA	2.61 ± 0.10 bcA	2.30 ± 0.11 cA	0.87 ± 0.02 dB
		KCl	2.60 ± 0.21 abAB	2.16 ± 0.15 bcABC	1.97 ± 0.09 cAB	1.66 ± 0.21 cA
		CaCl_2_	2.77 ± 0.22 aA	2.48 ± 0.08 aAB	1.74 ± 0.08 bB	1.77 ± 0.23 bA
		MgCl_2_	2.04 ± 0.06 bBC	1.73 ± 0.17 bC	1.19 ± 0.15 cC	0.69 ± 0.07 dB
		Mixed	1.82 ± 0.07 bC	1.89 ± 0.04 bBC	–	–
Dark	20.88 ± 0.71 aA	NaCl	17.53 ± 1.11 aA	17.92 ± 1.32 aA	16.88 ± 2.49 aA	8.52 ± 0.33 bA
		KCl	20.07 ± 1.06 aA	19.25 ± 1.90 aA	15.39 ± 2.20 bAB	2.14 ± 0.82 cB
		CaCl_2_	20.31 ± 0.26 aA	18.37 ± 1.27 aA	9.43 ± 0.68 bB	1.42 ± 0.17 cB
		MgCl_2_	19.53 ± 1.43 aA	7.14 ± 0.49 bB	5.03 ± 0.34 cC	1.72 ± 0.14 dB
		Mixed	11.34 ± 0.60 bB	9.18 ± 0.89 bB	–	–
			Radicle length (mm)			
Light–dark	33.11 ± 1.01 aA	NaCl	33.11 ± 0.68 aA	33.36 ± 0.91 aA	33.30 ± 2.73 aA	0.90 ± 0.08 bA
		KCl	28.36 ± 2.11 aAB	13.95 ± 1.36 bB	2.04 ± 0.14 cB	1.18 ± 0.07 dA
		CaCl_2_	33.20 ± 1.66 aAB	31.70 ± 2.77 aA	1.63 ± 0.18 bB	0.90 ± 0.12 cA
		MgCl_2_	24.45 ± 2.79 bA	1.92 ± 0.11 cC	1.42 ± 0.24 cdB	0.80 ± 0.12 dA
		Mixed	25.85 ± 2.38 bAB	2.46 ± 0.31 cC	–	–
Dark	16.24 ± 0.75 aB	NaCl	11.76 ± 1.16 bA	12.74 ± 1.31 abA	4.32 ± 0.54 cA	0.79 ± 0.06 dA
		KCl	14.95 ± 1.52 aA	6.03 ± 0.61 bB	2.93 ± 0.25 bcA	0.85 ± 0.12 cA
		CaCl_2_	14.63 ± 1.01 abA	11.22 ± 0.99 bA	3.89 ± 0.31 cA	0.87 ± 0.09 dA
		MgCl_2_	11.14 ± 0.56 bA	1.77 ± 0.15 cC	1.12 ± 0.13 cdB	0.67 ± 0.09 dA
		Mixed	2.39 ± 0.23 bB	3.42 ± 0.45 bBC	–	–
			Radicle/hypocotyl ratio			
Light–dark	11.86 ± 0.54 bA	NaCl	11.06 ± 0.24 bA	12.89 ± 0.68 abA	14.50 ± 0.90 aA	1.05 ± 0.12 cAB
		KCl	11.10 ± 0.91 aA	6.47 ± 0.43 bB	1.03 ± 0.04 cB	0.75 ± 0.21 cAB
		CaCl_2_	12.12 ± 0.75 aA	12.86 ± 1.28 aA	0.95 ± 0.14 bB	0.53 ± 0.12 bB
		MgCl_2_	12.07 ± 1.58 aA	1.16 ± 0.18 bC	1.26 ± 0.32 bB	1.16 ± 0.07 bA
		Mixed	14.21 ± 1.18 aA	1.30 ± 0.14 bC	–	–
Dark	0.78 ± 0.03 aB	NaCl	0.68 ± 0.08 aA	0.71 ± 0.03 aA	0.27 ± 0.04 bAB	0.09 ± 0.01 cB
		KCl	0.76 ± 0.10 aA	0.32 ± 0.04 bcC	0.20 ± 0.03 cB	0.56 ± 0.25 abA
		CaCl_2_	0.72 ± 0.05 aA	0.63 ± 0.10 abAB	0.42 ± 0.03 bA	0.62 ± 0.05 abA
		MgCl_2_	0.58 ± 0.04 bA	0.25 ± 0.03 cC	0.23 ± 0.04 cB	0.40 ± 0.05 bcA
		Mixed	0.21 ± 0.02 cB	0.37 ± 0.04 bBC	–	–
			Total length (mm)			
Light–dark	35.93 ± 0.98 aA	NaCl	36.10 ± 0.67 aA	35.97 ± 0.86 aA	35.60 ± 2.80 aA	1.77 ± 0.06 bB
		KCl	30.96 ± 2.22 aAB	16.11 ± 1.46 bB	4.02 ± 0.22 cB	2.84 ± 0.30 cA
		CaCl_2_	35.97 ± 1.79 aA	34.18 ± 2.74 aA	3.36 ± 0.16 bBC	2.66 ± 0.21 bA
		MgCl_2_	26.49 ± 2.76 bB	3.65 ± 0.10 cC	2.62 ± 0.16 cC	1.49 ± 0.19 dB
		Mixed	27.67 ± 2.41 bAB	4.35 ± 0.35 cC	–	–
Dark	37.12 ± 1.30 aA	NaCl	29.29 ± 1.70 bcA	30.66 ± 2.59 abA	21.21 ± 2.74 cA	9.31 ± 0.35 dA
		KCl	35.01 ± 1.55 aA	25.28 ± 2.18 bA	18.32 ± 2.26 bAB	2.99 ± 0.91 cB
		CaCl_2_	34.94 ± 1.10 abA	29.59 ± 0.91 bA	13.32 ± 0.88 cB	2.29 ± 0.24 dB
		MgCl_2_	30.67 ± 1.78 bA	8.91 ± 0.48 cB	6.15 ± 0.33 cC	2.39 ± 0.18 dB
		Mixed	13.73 ± 0.63 bB	12.60 ± 1.21 bB	–	–
			Seedling vigour index (% × cm)			
Light–dark	294.97 ± 8.03 aA	NaCl	332.95 ± 6.21 aA	289.02 ± 6.91 aA	161.67 ± 12.70 bA	2.56 ± 0.08 cB
		KCl	241.35 ± 17.33 bB	131.33 ± 11.88 cB	30.72 ± 1.66 dB	8.43 ± 0.90 eA
		CaCl_2_	301.00 ± 14.99 aAB	268.57 ± 21.52 aA	20.19 ± 0.99 bC	1.39 ± 0.11 cC
		MgCl_2_	236.14 ± 24.60 bB	31.70 ± 0.89 cC	10.64 ± 0.67 dD	0.20 ± 0.02 eD
		Mixed	117.58 ± 10.26 bC	3.80 ± 0.31 cD	–	–
Dark	305.01 ± 10.65 aA	NaCl	227.17 ± 13.21 bB	256.45 ± 21.65 abA	147.48 ± 19.05 cA	63.81 ± 2.38 dA
		KCl	318.59 ± 14.06 aA	227.10 ± 19.61 bA	162.09 ± 20.03 bA	19.15 ± 5.81 cB
		CaCl_2_	325.18 ± 10.28 aA	267.80 ± 8.27 aA	122.07 ± 8.11 bA	5.59 ± 0.60 cC
		MgCl_2_	289.91 ± 16.78 aA	84.86 ± 4.62 bB	51.35 ± 2.75 cB	5.85 ± 0.44 dC
		Mixed	121.25 ± 5.53 bC	84.45 ± 8.13 cB	–	–

Values are means ± standard error (SE). Different lowercase letters indicate significant differences among concentrations within the same salt type, light regime, and seedling parameter according to Tukey’s HSD test (*p* < 0.05). Different uppercase letters indicate significant differences among salt types within the same concentration, light regime, and seedling parameter according to Tukey’s HSD test (*p* < 0.05). For the control, uppercase letters indicate significant differences between light regimes. A dash (–) indicates that the parameter was not defined.

**Table 8:** Pearson correlations between selected germination/recovery parameters and the seedling vigour index of *D. viscosa*. Abbreviations: FGP_30_, final germination percentage at 30 days; GRI, germination rate index; T_50_, time to 50% of final germination; TGP_45_, total germination percentage after 30 d of salt treatment and 15 d of recovery; RP, recovery percentage; MGT*_recovery_*, mean germination time during recovery.

Germination/Recovery Parameter Correlated with Seedling Vigour Index	Global r	Light–Dark r	Dark r
FGP_30_	0.604 ***	0.657 ***	0.546 ***
GRI	0.686 ***	0.699 ***	0.788 ***
T_50_	−0.636 ***	−0.617 ***	−0.739 ***
TGP_45_	−0.016 ns	−0.044 ns	0.007 ns
RP	−0.643 ***	−0.678 ***	−0.695 ***
MGT*_recovery_*	0.060 ns	0.066 ns	−0.082 ns

Values are Pearson correlation coefficients (r). Global correlations were calculated using all treatments, whereas light–dark and dark correlations were calculated separately within each light regime. ns, not significant; ***, *p* < 0.001.

**Table 9:** Physicochemical characteristics of the salt solutions used in the germination experiment. C, nominal total salt concentration; Cl^−^, chloride concentration; I, ionic strength; EC, electrical conductivity; Ψs, estimated osmotic potential. Ionic strength was calculated as I = 1/2 ∑ c_i_ z_i_^2^, where c_i_ is the molar concentration of ion i and z_i_ is its charge. Osmotic potential was estimated using the van ’t Hoff equation (Ψs = −iCRT) at 25 °C, where i is the van ’t Hoff factor, C is the molar concentration, R is the universal gas constant, and T is the absolute temperature.

Salt	C (mM)	Cl^−^ (mM)	I (mM)	EC (mS cm^−1^)	pH	Ψs (MPa)
Control (Distilled Water)	0	0	0	0.0	7.28	0.000
NaCl	22.5	22.5	22.5	1.7	7.42	−0.112
	45	45	45	3.2	7.25	−0.223
	90	90	90	6.8	6.99	−0.446
	180	180	180	13.5	7.15	−0.892
	360	360	360	25.3	6.84	−1.785
KCl	22.5	22.5	22.5	2.0	7.47	−0.112
	45	45	45	3.9	7.31	−0.223
	90	90	90	7.8	7.11	−0.446
	180	180	180	15.5	6.94	−0.892
	360	360	360	32.3	6.57	−1.785
CaCl_2_	22.5	45	67.5	3.3	7.74	−0.167
	45	90	135	6.5	7.34	−0.335
	90	180	270	12.6	7.02	−0.669
	180	360	540	23.1	6.80	−1.339
	360	720	1080	45.6	6.46	−2.677
MgCl_2_	22.5	45	67.5	3.3	7.50	−0.167
	45	90	135	5.9	7.33	−0.335
	90	180	270	11.7	7.28	−0.669
	180	360	540	22.1	7.06	−1.339
	360	720	1080	40.0	6.44	−2.677
Mixed	22.5	33.75	45	2.4	7.21	−0.139
	45	67.5	90	5.0	7.01	−0.279
	90	135	180	9.7	6.76	−0.558
	180	270	360	18.5	6.68	−1.116
	360	540	720	35.2	6.61	−2.231

**Table 10:** Germination and recovery parameters calculated from daily germination counts. Abbreviations: n_i_, number of seeds germinated on day or during interval i; N, total number of seeds used in the calculation; G_i_ and G_j_, cumulative germination percentages at counts i and j, respectively; G_j_ − G_j−1_, percentage of seeds germinated between two consecutive counts; D_j_, number of days elapsed from sowing to count j; D, total assay duration; FGP, final germination percentage at the end of the 30-day assay (FGP_30_); g_i_, percentage of seeds germinated on day i; t_i_, time elapsed from sowing to count i; Nf, final number of germinated seeds; N_i_ and N_j_, cumulative numbers of seeds germinated immediately before and after reaching Nf/2, respectively; t_i_ and t_j_, the corresponding times; f_i_, relative frequency of germination on day i, calculated as n_i_/Σn_i_; C(x, 2) = x(x − 1)/2, number of possible pairs among x seeds; C(n_i_, 2), number of pairs among seeds germinating on the same day; C(Σn_i_, 2), total number of possible pairs among all germinated seeds; N_r_, number of seeds germinated during the recovery phase; N_ng_, number of ungerminated seeds transferred to the recovery assay; N_g_, number of seeds germinated during the salt-treatment phase; n_ri_, number of seeds germinated on recovery day i; and t_ri_, time elapsed from the beginning of the recovery phase.

Parameter	Abbreviation	Equation	Interpretation	References
Germination percentage	FGP1_4,_ FGP_21_, FGP_30_	FGP = (Σn_i_/N) × 100	Cumulative germination by 14, 21, or 30 days	[7,18]
Modified Timson index	GV*_Timson_*	GV*_Timson_* = ΣG_i_/D	Integrated measure of germination extent and rate	[7,77]
Peak value	PV	PV = max(G_i_/t_i_)	Maximum cumulative germination rate	[18,78]
Mean daily germination	MDG	MDG = FGP/D	Mean germination percentage per day	[18,78]
Czabator germination value	GV*_Czabator_*	GV*_Czabator_* = PV × MDG	Composite measure of germination extent and rate	[18,78]
Coefficient of velocity of germination	CVG	CVG = [Σn_i_/Σ(n_i_ × t_i_)] × 100	Relative germination rate	[18,79]
Germination rate index	GRI	GRI = Σ(g_i_/t_i_)	Germination rate over time	[18,79]
Mean germination time	MGT	MGT = Σ(n_i_ × t_i_)/Σn_i_	Mean time to germination	[80]
Time to 50% of final germination	T_50_	T_50_ = t_i_ + [(Nf/2 − N_i_)(t_j_ − t_i_)]/(N_j_ − N_i_)	Time required to reach 50% of final germination	[7,81]
Uncertainty	U	U = −Σf_i_ log_2_(f_i_)	Temporal spread of germination	[7,17,82]
Synchrony	Z	Z = ΣC(n_i_, 2)/C(Σn_i_, 2); C(n_i_, 2) = n_i_(n_i_ − 1)/2	Degree of simultaneous germination	[7,17,82]
Recovery percentage	RP	RP = (N_r_/N_ng_) × 100	Germination after transfer to non-saline medium	[12,83]
Total germination percentage	TGP_45_	TGP_45_ = [(N_g_ + N_r_)/N] × 100	Cumulative germination after treatment and recovery	[12]
Mean germination time during recovery	MGT*_recovery_*	MGT*_recovery_* = Σ(n_ri_ × t_ri_)/Σn_ri_	Mean time to germination during recovery	[12,80]

## Data Availability

The original contributions presented in this study are included in the article and its Supplementary Materials. Further inquiries can be directed to the corresponding author.

## Supplementary Materials

The following supporting information can be downloaded at https://www.mdpi.com/article/10.3390/plants15172607/s1, Table S1: Two-way ANOVAs for the effect of salt type, salt concentration, and their interaction on seed germination and recovery parameters of *D. viscosa*, analysed separately under the light–dark regime and continuous darkness; Table S2: Final germination percentages of *D. viscosa* seeds after 14 and 21 days of exposure to different salt types and concentrations under the light–dark regime and continuous darkness; Table S3: Additional germination parameters of *D. viscosa* seeds under different salt types and concentrations under the light–dark regime and continuous darkness; Table S4: Analysis of variance and Tukey’s HSD test for comparing the slopes of the relationships between germination rate (1/T_50_) and Cl^−^ concentration in *D. viscosa*; Table S5: Two-way ANOVAs for the effects of salt type, salt concentration, and their interaction on seedling growth parameters of *D. viscosa*, analysed separately under the light–dark regime and continuous darkness; Table S6: Eigenvalues, explained variance, and variable loadings for principal component analyses.

## Abbreviations

The following abbreviations are used in this manuscript:

ANOVA	Analysis of variance
CVG	Coefficient of velocity of germination
FGP	Final germination percentage
GRI	Germination rate index
GV_*Czabator*_	Czabator germination value
GV_*Timson*_	Modified Timson index
HL	Hypocotyl length
MDG	Mean daily germination
MGT	Mean germination time
PCA	Principal component analysis
PV	Peak value
R/H	Radicle-to-hypocotyl length ratio
RL	Radicle length
RP	Recovery percentage
SVI	Seedling vigour index
T_50_	Time to 50% of final germination
TGP	Total germination percentage
TL	Total seedling length
Tukey’s HSD	Tukey’s honestly significant difference test
U	Uncertainty
Z	Synchrony
