# Supplementary material for: Chloride Salts and Light Modulate Germination and Early Seedling Performance in Dittrichia viscosa
Source: Plants (Basel). 2026 Aug 26;15(17):2607. doi: 10.3390/plants15172607 (PMC13567514; doi:10.3390/plants15172607)
Supplement: Supplementary file 1 [file plants-15-02607-s001.zip › plants-4480576-supplementary.pdf]

Supplementary Materials.

Table S1. Two-way ANOVA parameters in relation to the effect of salt type, salt concentration, and their interaction on seed germination and recovery parameters of *D. viscosa*, analysed separately under the light–dark regime and continuous darkness. Abbreviations: PV, peak value; GV_{Timson}, Modified Timson index; $GV_{Czabator}$, germination value; CVG, coefficient of velocity of germination; GRI, germination rate index; MGT, mean germination time; T_{50}, time to 50% germination; Z, synchrony; U, uncertainty; RP, recovery percentage; $MGT_{recovery}$, mean germination time during recovery.

Parameter	Salt (S)		Concentration (C)		S × C	
	d.f.	F-ratio	d.f.	F-ratio	d.f.	F-ratio
PV (light)	4	21.01***	5	213.62***	20	4.78***
PV (dark)	4	13.77***	5	148.15***	20	2.95***
GV_{Timson} (light)	4	81.50***	5	498.79***	20	24.29***
GV_{Timson} (dark)	4	27.02***	5	245.29***	20	8.81***
$GV_{Czabator}$ (light)	4	15.49***	5	145.93***	20	9.59***
$GV_{Czabator}$ (dark)	4	10.78***	5	104.37***	20	2.64***
CVG (light)	4	14.80***	4	108.63***	16	7.66***
CVG (dark)	4	9.58***	4	88.46***	16	6.93***
GRI (light)	4	35.75***	5	379.83***	20	8.78***
GRI (dark)	4	15.04***	5	161.02***	20	3.66***
MGT (light)	4	62.36***	4	239.52***	16	67.39***
MGT (dark)	4	37.76***	4	243.71***	16	54.91***
T_{50} (light)	4	69.72***	4	219.80***	16	62.97***
T_{50} (dark)	4	62.72***	4	409.68***	16	88.16***
Z (light)	4	1.32 ns	4	19.68***	16	3.21***
Z (dark)	4	3.88**	4	42.08***	16	6.00***
U (light)	4	26.60***	4	177.01***	16	43.22***
U (dark)	4	5.03***	4	3.81**	16	8.45***
RP (light)	4	17.22***	4	62.41***	16	27.22***
RP (dark)	4	78.92***	4	295.05***	16	77.07***
$MGT_{recovery}$ (light)	4	93.14***	4	48.33***	16	25.78***
$MGT_{recovery}$ (dark)	4	23.91***	4	39.57***	16	11.31***

ns, not significant; *, $p < 0.05$; **, $p < 0.01$; ***, $p < 0.001$. Degrees of freedom vary because undefined observations were excluded from the corresponding analyses.

Table S2. Final germination percentages of *D. viscosa* seeds after 14 (FGP_{14}) and 21 days (FGP_{21}) of exposure to NaCl, KCl, $CaCl_2$, $MgCl_2$ and their equimolar mixture at increasing concentrations under two light regimes, analysed using binomial generalised linear models.

Light regime	Control	Salt	Concentration (mM)				
			22.5	45	90	180	360
			**FGP_{14} (%)**				
Light–dark	80.25 ± 1.59 aA	NaCl	82.33 ± 5.84 aA	63.71 ± 6.93 aA	34.17 ± 3.44 bB	0.00 ± 0.00 cA	0.00 ± 0.00 cA
		KCl	74.44 ± 7.38 aA	69.66 ± 4.70 aA	64.15 ± 3.83 aA	18.32 ± 1.31 bA	0.00 ± 0.00 bA
		$CaCl_2$	62.64 ± 2.44 aA	63.31 ± 6.05 aA	42.98 ± 4.24 bA	0.00 ± 0.00 cA	0.00 ± 0.00 cA
		$MgCl_2$	70.41 ± 4.48 aA	77.82 ± 3.37 aA	27.50 ± 2.47 bB	0.00 ± 0.00 cA	0.00 ± 0.00 cA
		Mixed	19.53 ± 1.87 bB	0.00 ± 0.00 cB	0.00 ± 0.00 cC	0.00 ± 0.00 cA	0.00 ± 0.00 cA
Dark	80.37 ± 3.99 aA	NaCl	77.56 ± 6.34 aA	83.63 ± 7.90 aA	67.26 ± 7.25 aA	44.98 ± 3.90 bA	0.00 ± 0.00 cA
		KCl	88.05 ± 4.16 aA	88.18 ± 5.83 aA	87.15 ± 4.03 aA	34.12 ± 3.11 bA	0.00 ± 0.00 cA
		$CaCl_2$	90.28 ± 5.73 aA	86.61 ± 4.94 aA	82.59 ± 6.37 aA	6.79 ± 4.26 bB	0.00 ± 0.00 bA
		$MgCl_2$	94.53 ± 0.85 aA	95.26 ± 1.96 aA	78.63 ± 4.43 bA	6.55 ± 4.05 cB	0.00 ± 0.00 cA
		Mixed	84.85 ± 5.01 aA	51.51 ± 4.16 bB	0.00 ± 0.00 cB	0.00 ± 0.00 cB	0.00 ± 0.00 cA
			**FGP_{21} (%)**				
Light–dark	82.11 ± 1.90 aA	NaCl	89.78 ± 4.48 aA	72.43 ± 5.40 aA	40.83 ± 4.00 bB	4.17 ± 0.46 cA	0.00 ± 0.00 cA
		KCl	82.36 ± 5.81 aA	74.24 ± 7.34 aA	72.91 ± 7.23 aA	27.79 ± 1.34 bA	0.00 ± 0.00 cA
		$CaCl_2$	82.20 ± 5.87 aA	78.57 ± 5.50 aA	54.63 ± 4.93 bA	0.00 ± 0.00 cB	0.00 ± 0.00 cA
		$MgCl_2$	87.05 ± 2.92 aA	83.32 ± 3.75 aA	31.07 ± 2.98 bB	0.00 ± 0.00 cB	0.00 ± 0.00 cA
		Mixed	35.08 ± 1.80 bB	4.30 ± 0.30 cB	0.00 ± 0.00 cC	0.00 ± 0.00 cB	0.00 ± 0.00 cA
Dark	81.74 ± 3.85 aA	NaCl	77.56 ± 6.34 aA	83.63 ± 7.90 aA	69.53 ± 6.20 aB	64.71 ± 4.78 aA	0.00 ± 0.00 bA
		KCl	89.52 ± 3.73 aA	89.85 ± 5.92 aA	87.15 ± 4.03 aA	55.21 ± 5.14 bA	0.00 ± 0.00 cA
		$CaCl_2$	93.06 ± 4.17 aA	86.61 ± 4.94 aA	91.67 ± 5.00 aA	16.88 ± 1.30 bB	0.00 ± 0.00 cA
		$MgCl_2$	94.53 ± 0.85 aA	95.26 ± 1.96 aA	81.13 ± 4.14 bA	17.90 ± 1.75 cB	0.00 ± 0.00 dA
		Mixed	86.04 ± 5.57 aA	61.77 ± 5.90 bB	0.00 ± 0.00 cC	0.00 ± 0.00 cC	0.00 ± 0.00 cA

Values are means ± SE. Different lowercase letters indicate significant differences among concentrations within the same salt type and light regime, whereas different uppercase letters indicate significant differences among salt types within the same concentration and light regime, based on Tukey-adjusted pairwise contrasts from binomial generalised linear models ($p < 0.05$). For the control, uppercase letters indicate significant differences between light regimes.

Table S3. Additional germination parameters of *D. viscosa* seeds under the light–dark regime and continuous darkness, across different salt types and salt concentrations.

Light regime	Control	Salt	Concentration (mM)				
			22.5	45	90	180	360
**PV**							
Light–dark	9.47 ± 0.59 aB	NaCl	9.35 ± 0.93 aA	5.36 ± 0.50 bA	3.15 ± 0.28 bcB	0.61 ± 0.05 cB	0.00 ± 0.00 cA
		KCl	7.61 ± 0.65 abAB	6.62 ± 0.48 bA	5.80 ± 0.49 bA	2.08 ± 0.16 cA	0.00 ± 0.00 cA
		$CaCl_2$	6.05 ± 0.34 bB	4.97 ± 0.42 bA	3.46 ± 0.37 bB	0.18 ± 0.03 cC	0.00 ± 0.00 cA
		$MgCl_2$	6.35 ± 0.75 bB	6.05 ± 0.37 bA	2.23 ± 0.33 cB	0.04 ± 0.02 cC	0.00 ± 0.00 cA
		Mixed	2.04 ± 0.24 bC	0.32 ± 0.06 bB	0.00 ± 0.00 bC	0.00 ± 0.00 bC	0.00 ± 0.00 bA
Dark	14.23 ± 1.02 aA	NaCl	12.53 ± 1.33 abAB	10.62 ± 0.94 abB	7.68 ± 0.68 bcA	4.08 ± 0.42 cdA	0.00 ± 0.00 dA
		KCl	15.60 ± 1.34 aA	14.65 ± 1.21 aA	10.12 ± 0.78 aA	3.11 ± 0.37 bA	0.00 ± 0.00 bA
		$CaCl_2$	12.37 ± 1.01 abAB	9.87 ± 0.93 abB	7.64 ± 0.82 bA	1.02 ± 0.15 cB	0.00 ± 0.00 cA
		$MgCl_2$	16.27 ± 1.62 aA	11.86 ± 0.45 abAB	7.79 ± 0.67 bA	1.00 ± 0.08 cB	0.00 ± 0.00 cA
		Mixed	10.17 ± 0.28 aB	4.19 ± 0.38 bC	0.05 ± 0.03 bB	0.00 ± 0.00 bB	0.00 ± 0.00 bA
**GV_{Timson}**							
Light–dark	67.12 ± 1.68 aA	NaCl	70.51 ± 4.95 aA	49.03 ± 4.56 bA	27.96 ± 2.58 cB	3.80 ± 0.54 dB	0.00 ± 0.00 dA
		KCl	62.64 ± 5.76 aA	58.70 ± 4.91 aA	53.92 ± 4.49 aA	18.34 ± 0.87 bA	0.00 ± 0.00 cA
		$CaCl_2$	57.04 ± 4.68 abA	52.37 ± 5.50 bA	34.07 ± 3.98 cB	0.42 ± 0.05 dC	0.00 ± 0.00 dA
		$MgCl_2$	63.08 ± 4.69 aA	60.06 ± 3.70 aA	21.97 ± 2.11 bB	0.06 ± 0.04 cC	0.00 ± 0.00 cA
		Mixed	22.01 ± 1.64 bB	2.17 ± 2.01 cB	0.00 ± 0.00 cD	0.00 ± 0.00 cC	0.00 ± 0.00 cA
Dark	72.03 ± 3.64 aA	NaCl	67.11 ± 6.04 aA	68.94 ± 5.50 aA	55.17 ± 4.74 abB	41.63 ± 4.30 bA	0.00 ± 0.00 cA
		KCl	78.82 ± 3.86 aA	78.23 ± 5.52 aA	71.82 ± 3.93 aA	35.31 ± 3.63 bA	0.00 ± 0.00 cA
		$CaCl_2$	77.94 ± 4.37 aA	71.14 ± 4.15 aA	67.89 ± 4.70 aAB	10.02 ± 3.58 bB	0.00 ± 0.00 bA
		$MgCl_2$	82.89 ± 1.13 aA	79.67 ± 1.45 aA	64.58 ± 3.14 aAB	10.15 ± 1.44 bB	0.00 ± 0.00 bA
		Mixed	71.13 ± 2.78 aA	42.77 ± 3.71 bB	0.34 ± 0.09 cC	0.00 ± 0.00 cB	0.00 ± 0.00 cA
**$GV_{Czabator}$**							
Light–dark	26.05 ± 1.89 aB	NaCl	28.42 ± 1.96 aA	14.43 ± 0.78 bBC	4.90 ± 0.75 cBC	0.32 ± 0.06 cB	0.00 ± 0.00 cA
		KCl	21.62 ± 2.99 abAB	18.54 ± 0.91 abA	14.99 ± 0.89 bA	2.14 ± 0.46 cA	0.00 ± 0.00 cA
		$CaCl_2$	17.28 ± 1.65 bB	13.38 ± 0.62 bC	7.75 ± 0.82 bcB	0.04 ± 0.02 cB	0.00 ± 0.00 cA
		$MgCl_2$	19.29 ± 1.86 abB	17.67 ± 1.23 bAB	4.06 ± 0.85 cC	0.01 ± 0.01 cB	0.00 ± 0.00 cA
		Mixed	3.07 ± 0.42 bC	0.25 ± 0.16 bD	0.00 ± 0.00 bD	0.00 ± 0.00 bB	0.00 ± 0.00 bA
Dark	40.08 ± 3.60 aA	NaCl	33.54 ± 4.73 abAB	31.49 ± 3.02 abB	19.21 ± 0.92 bcB	9.66 ± 0.86 cA	0.00 ± 0.00 cA
		KCl	47.61 ± 4.44 aAB	44.44 ± 3.60 aA	30.20 ± 2.30 aA	6.96 ± 0.48 bB	0.00 ± 0.00 bA
		$CaCl_2$	38.74 ± 3.60 abAB	29.90 ± 2.06 abB	23.88 ± 3.11 bAB	0.96 ± 0.10 cC	0.00 ± 0.00 cA
		$MgCl_2$	51.23 ± 5.01 aA	37.66 ± 1.66 abAB	21.85 ± 1.89 bAB	1.09 ± 0.18 cC	0.00 ± 0.00 cA
		Mixed	29.92 ± 1.52 aB	9.99 ± 0.82 bC	0.01 ± 0.01 bC	0.00 ± 0.00 bC	0.00 ± 0.00 bA
**CVG**							
Light–dark	15.81 ± 0.69 aB	NaCl	14.43 ± 1.72 aA	8.66 ± 0.87 bA	8.08 ± 0.78 bAB	4.71 ± 0.52 bB	–

Light regime	Control	Salt	Concentration (mM)				
			22.5	45	90	180	360
		KCl	12.48 ± 1.19 abA	10.64 ± 0.78 bA	10.34 ± 0.79 bA	9.10 ± 0.97 bA	–
		$CaCl_2$	10.27 ± 1.01 bAB	9.20 ± 0.30 bA	7.39 ± 0.45 bcB	3.54 ± 0.16 cB	–
		$MgCl_2$	10.38 ± 0.76 bAB	9.76 ± 0.09 bcA	6.39 ± 0.72 bcB	3.33 ± 0.00 cB	–
		Mixed	6.78 ± 0.50 bB	4.00 ± 0.22 bB	–	–	–
Dark	21.70 ± 1.16 aA	NaCl	19.79 ± 0.96 abAB	15.60 ± 1.55 bB	13.90 ± 0.96 bcAB	7.99 ± 0.63 cA	–
		KCl	21.00 ± 2.03 abA	21.29 ± 1.82 abA	15.10 ± 0.45 bA	6.88 ± 0.49 cAB	–
		$CaCl_2$	17.21 ± 1.17 abAB	13.91 ± 1.45 bB	11.38 ± 0.48 bcB	5.18 ± 0.50 cB	–
		$MgCl_2$	21.52 ± 1.23 aA	17.01 ± 0.65 abAB	12.98 ± 0.87 bAB	5.59 ± 0.50 cAB	–
		Mixed	14.91 ± 1.07 bB	8.07 ± 0.33 cC	4.55 ± 0.00 bcC	–	–
**MGT (days)**							
Light–dark	6.47 ± 0.30 cA	NaCl	7.47 ± 0.80 cB	12.03 ± 1.20 bB	12.73 ± 1.24 bB	22.25 ± 1.93 aA	–
		KCl	8.30 ± 0.77 abB	9.56 ± 0.73 aB	9.87 ± 0.86 aB	11.41 ± 1.35 aB	–
		$CaCl_2$	10.19 ± 0.75 cB	10.91 ± 0.35 cB	13.68 ± 0.83 bAB	28.33 ± 1.20 aA	–
		$MgCl_2$	9.82 ± 0.82 cB	10.25 ± 0.10 cB	18.17 ± 1.50 bA	30.00 ± 0.00 aA	–
		Mixed	14.98 ± 1.09 bA	25.50 ± 1.43 aA	–	–	–
Dark	4.78 ± 0.31 cB	NaCl	5.09 ± 0.25 bcB	6.61 ± 0.33 bcBC	7.29 ± 0.46 bBC	12.73 ± 0.94 aB	–
		KCl	5.00 ± 0.37 bB	4.88 ± 0.24 bC	6.64 ± 0.20 bC	14.76 ± 1.12 aAB	–
		$CaCl_2$	5.90 ± 0.46 bcAB	7.41 ± 0.70 bcB	8.83 ± 0.36 bB	20.21 ± 2.57 aA	–
		$MgCl_2$	4.69 ± 0.26 cB	5.90 ± 0.22 bcBC	7.83 ± 0.60 bBC	18.16 ± 1.53 aAB	–
		Mixed	6.80 ± 0.45 cA	12.46 ± 0.55 bA	22.00 ± 0.00 aA	–	–
**Z**							
Light–dark	0.10 ± 0.01 aB	NaCl	0.10 ± 0.03 aA	0.04 ± 0.01 abB	0.03 ± 0.02 abAB	0.00 ± 0.00 bA	–
		KCl	0.06 ± 0.01 abAB	0.03 ± 0.00 bB	0.05 ± 0.01 abAB	0.00 ± 0.00 bA	–
		$CaCl_2$	0.04 ± 0.01 bB	0.02 ± 0.01 bB	0.08 ± 0.01 abA	–	–
		$MgCl_2$	0.04 ± 0.01 bcAB	0.10 ± 0.01 abA	0.02 ± 0.01 cB	–	–
		Mixed	0.00 ± 0.00 bB	0.00 ± 0.00 abB	–	–	–
Dark	0.22 ± 0.02 abA	NaCl	0.26 ± 0.02 aA	0.13 ± 0.01 bcBC	0.08 ± 0.01 cB	0.05 ± 0.01 cA	–
		KCl	0.22 ± 0.02 abAB	0.25 ± 0.02 aA	0.12 ± 0.01 bcA	0.03 ± 0.01 cAB	–
		$CaCl_2$	0.21 ± 0.02 abAB	0.16 ± 0.01 abB	0.10 ± 0.01 bcAB	0.02 ± 0.01 cB	–
		$MgCl_2$	0.25 ± 0.02 aA	0.19 ± 0.01 abAB	0.10 ± 0.01 bcAB	0.02 ± 0.01 cAB	–
		Mixed	0.14 ± 0.01 abB	0.09 ± 0.00 bC	–	–	–
**U**							
Light–dark	2.58 ± 0.09 aA	NaCl	2.64 ± 0.29 aA	2.13 ± 0.14 abB	1.77 ± 0.11 bB	0.25 ± 0.25 cB	–
		KCl	2.75 ± 0.07 aA	2.81 ± 0.07 aA	2.91 ± 0.08 aA	0.95 ± 0.05 bA	–
		$CaCl_2$	2.79 ± 0.34 aA	2.92 ± 0.21 aA	1.94 ± 0.11 bB	0.00 ± 0.00 cB	–
		$MgCl_2$	3.16 ± 0.10 aA	2.68 ± 0.19 abAB	1.83 ± 0.10 cB	0.00 ± 0.00 dB	–
		Mixed	2.30 ± 0.23 aA	0.79 ± 0.17 bC	–	–	–
Dark	2.08 ± 0.11 aB	NaCl	1.71 ± 0.15 aB	1.82 ± 0.14 aC	2.15 ± 0.15 aB	2.40 ± 0.23 aA	–
		KCl	2.11 ± 0.20 aAB	1.98 ± 0.19 aBC	2.55 ± 0.05 aAB	2.65 ± 0.27 aA	–
		$CaCl_2$	2.36 ± 0.13 abAB	2.59 ± 0.13 abAB	2.87 ± 0.19 aA	1.86 ± 0.30 bA	–
		$MgCl_2$	2.19 ± 0.22 bAB	2.41 ± 0.09 abABC	2.94 ± 0.14 aA	2.55 ± 0.14 abA	–
		Mixed	2.65 ± 0.06 abA	3.04 ± 0.22 aA	0.00 ± 0.00 cC	–	–

Values are means ± SE. Different lowercase letters indicate significant differences among concentrations within the same salt type and light regime according to Tukey's HSD test ($p < 0.05$). Different uppercase letters indicate significant differences among salt types within the same concentration and light regime according to Tukey's HSD test ($p < 0.05$). For the control, uppercase letters indicate significant differences between light regimes. A dash (–) indicates that the parameter was not defined.

Table S4. Analysis of variance and Tukey's HSD test for comparing the slopes of the relationships between germination rate ($1/T_{50}$) and Cl^- concentration in *D. viscosa*. Slopes are means ± SE.

Global effects on the relationship between $1/T_{50}$ and Cl^- concentration								
Parameter	Cl^- concentration		S × Cl^-		L × Cl^-		S × L × Cl^-	
	d.f.	F-ratio	d.f.	F-ratio	d.f.	F-ratio	d.f.	F-ratio
$1/T_{50}$ (d^{-1})	1	368.51***	4	27.64***	1	8.72**	4	3.27*

Comparison of slopes among salt types and between light regimes				
Salt	Light–dark	Continuous darkness	L × Cl^-	
	Slope ± SE (Tukey group)	Slope ± SE (Tukey group)	d.f.	F-ratio
NaCl	−0.000727 ± 0.000096 b	−0.001087 ± 0.000102 b	1	0.89 ns
KCl	−0.000360 ± 0.000096 bc	−0.000985 ± 0.000102 b	1	15.56**
$CaCl_2$	−0.000423 ± 0.000053 c	−0.000682 ± 0.000051 c	1	3.99 ns
$MgCl_2$	−0.000515 ± 0.000071 bc	−0.000614 ± 0.000056 c	1	0.17 ns
Mixed	−0.002039 ± 0.000253 a	−0.002174 ± 0.000202 a	1	0.67 ns

ns, not significant; *, $p < 0.05$; **, $p < 0.01$; ***, $p < 0.001$. Within each light regime, slopes followed by different letters differ significantly among salt types according to Tukey's HSD test ($p < 0.05$).

Table S5. Two-way ANOVA parameters in relation to the effects of salt type, salt concentration, and their interaction on seedling growth parameters of *D. viscosa*, analysed separately under the light–dark regime and continuous darkness. Abbreviations: H, hypocotyl length; R, radicle length; R/H, radicle/hypocotyl ratio; TL, total length; SVI, seedling vigour index. The 360 mM treatment was excluded because no measurable seedlings were obtained.

Parameter	Salt (S)		Concentration (C)		S × C	
	d.f.	F-ratio	d.f.	F-ratio	d.f.	F-ratio
H (light–dark)	4	49.06***	4	224.22***	16	16.72***
H (dark)	4	74.00***	4	498.30***	16	23.77***
R (light–dark)	4	157.06***	4	1053.01***	16	86.08***
R (dark)	4	51.16***	4	511.23***	16	27.36***
R/H (light–dark)	4	106.16***	4	816.70***	16	81.93***
R/H (dark)	4	17.39***	4	97.40***	16	15.78***
TL (light–dark)	4	157.51***	4	1033.15***	16	81.41***
TL (dark)	4	67.86***	4	528.88***	16	21.45***
SVI (light–dark)	4	215.64***	4	1527.01***	16	97.12***
SVI (dark)	4	57.50***	4	537.98***	16	19.93***

ns, not significant; *, $p < 0.05$; **, $p < 0.01$; ***, $p < 0.001$.

Table S6. Eigenvalues, explained variance and variable loadings for the principal component analyses. Abbreviations: FGP_{30}, final germination percentage at 30 days; GRI, germination rate index; T_{50}, time to 50% of final germination; TGP_{45}, total germination percentage after 30 d of salt treatment and 15 d of recovery; RP, recovery percentage; $MGT_{recovery}$, mean germination time during recovery; HL, hypocotyl length; RL, radicle length; R/H, radicle/hypocotyl ratio; TL, total length; SVI, seedling vigour index.

Eigenvalues and explained variance				
PCA	Component	Eigenvalue	Variance (%)	Cumulative variance (%)
Integrated PCA	PC1	5.163	46.93	46.93
	PC2	2.745	24.96	71.89
	PC3	1.188	10.80	82.70
Germination–recovery PCA	PC1	3.567	59.46	59.46
	PC2	1.153	19.21	78.67
	PC3	0.945	15.75	94.42
Recovery PCA	PC1	1.821	60.69	60.69

	PC2	0.959	31.96	92.65
	PC3	0.221	7.35	100.00
Seedling PCA	PC1	3.304	66.08	66.08
	PC2	1.624	32.47	98.56
	PC3	0.058	1.15	99.71
**Variable loadings**				
**PCA**	**Variable**	**PC1**	**PC2**	**PC3**
Integrated PCA	FGP_{30}	0.872	-0.349	-0.189
	GRI	0.823	-0.476	-0.172
	T_{50}	-0.831	0.363	0.038
	TGP_{45}	0.150	-0.137	-0.920
	RP	-0.753	0.404	-0.289
	$MGT_{recovery}$	-0.089	0.331	-0.361
	HL	0.477	-0.613	0.221
	RL	0.659	0.747	0.026
	R/H	0.533	0.823	0.007
	TL	0.825	0.505	0.107
	SVI	0.901	0.329	0.013
Germination–recovery PCA	FGP_{30}	0.969	0.110	-0.024
	GRI	0.964	0.129	-0.039
	T_{50}	-0.938	0.015	-0.037
	TGP_{45}	0.150	0.929	0.314
	RP	-0.864	0.306	0.253
	$MGT_{recovery}$	-0.223	0.409	-0.883
Recovery PCA	TGP_{45}	0.939	-0.072	-0.335
	RP	0.357	0.931	0.075
	$MGT_{recovery}$	-0.901	0.294	-0.320
Seedling PCA	HL	0.254	0.960	-0.119
	RL	0.944	-0.322	0.008
	R/H	0.750	-0.649	-0.106
	TL	0.971	0.227	-0.056
	SVI	0.918	0.356	0.170
